# Arabidopsis BRUTUS-LIKE E3 ligases negatively regulate iron uptake by targeting transcription factor FIT for recycling

**DOI:** 10.1073/pnas.1907971116

**Published:** 2019-08-14

**Authors:** Jorge Rodríguez-Celma, James M. Connorton, Inga Kruse, Robert T. Green, Marina Franceschetti, Yi-Tze Chen, Yan Cui, Hong-Qing Ling, Kuo-Chen Yeh, Janneke Balk

**Affiliations:** ^a^Department of Biological Chemistry, John Innes Centre, Norwich NR4 7UH, United Kingdom;; ^b^School of Biological Sciences, University of East Anglia, Norwich NR4 7TJ, United Kingdom;; ^c^Agricultural Biotechnology Research Center, Academia Sinica, Taipei 11529, Taiwan;; ^d^Institute of Genetics and Developmental Biology, Chinese Academy of Sciences, Beijing 100101, China

**Keywords:** iron, ubiquitin, bHLH transcription factor, dicotyledon, micronutrient

## Abstract

Organisms need to balance sufficient uptake of iron (Fe) with possible toxicity. In plant roots, a regulon of uptake genes is transcriptionally activated under Fe deficiency, but it is unknown how this response is inactivated when Fe becomes available. Here we describe the function of 2 partially redundant E3 ubiquitin ligases, BRUTUS-LIKE1 (BTSL1) and BTSL2, in *Arabidopsis thaliana* and provide evidence that they target the transcription factor FIT, a key regulator of Fe uptake, for degradation. The *btsl* double mutant failed to effectively down-regulate the transcription of genes controlled by FIT, and accumulated toxic levels of Fe in roots and leaves. The C-terminal domains of BTSL1 and BTSL2 exhibited E3 ligase activity, and interacted with FIT but not its dimeric partner bHLH39. The BTSL proteins were able to poly-ubiquitinate FIT in vitro and promote FIT degradation in vivo. Thus, posttranslational control of FIT is critical to prevent excess Fe uptake.

## Significance

Mechanisms to balance the acquisition of sufficient Fe while preventing a toxic overload differ in bacteria, fungi, animals, and plants. Identification of specific E3 ligases acting directly on a key transcription factor for Fe uptake in Arabidopsis indicates how this balance is regulated in dicotyledonous plants. The domain structure and function of the E3 ligases show interesting parallels to a distantly related protein regulating Fe homeostasis in mammals. Moreover, the accumulation of Fe in weaker mutant alleles of the E3 ligases could be exploited for biofortification of crops.

Iron (Fe) is the fourth most abundant element in the Earth’s crust, but its bioavailability is greatly limited by the insolubility of Fe hydroxides. High-affinity uptake mechanisms are therefore essential for most organisms, from prokaryotes to multicellular species. Plants have developed 2 molecular strategies for Fe uptake, historically divided into Strategy I (reductive strategy) present in dicotyledonous plants, and Strategy II (chelating strategy) in grasses (1, 2). The reductive strategy in plants such as *Arabidopsis thaliana* involves a ferric reductive oxidase, FRO2, to reduce Fe^3+^ to Fe^2+^, which is then taken up by the iron-regulated transporter IRT1. A key regulator of Fe uptake in dicot plants is the basic helix–loop–helix (bHLH) transcription factor FIT (FER-like iron deficiency-induced transcription factor) (3). FIT forms heterodimers with 1 of 4 bHLH proteins from subgroup Ib; namely, bHLH38, bHLH39, bHLH100, and bHLH101. Mutant studies combined with transcriptomics have identified more than 400 genes that are controlled by FIT (4, 5). These include *FRO2* and *IRT1* for which direct promoter binding by the FIT-bHLH Ib dimer has been shown (6, 7).

Fe is essential as a cofactor for many enzymes, but in its free form it is toxic. Redox chemistry of Fe^2+^/Fe^3+^ catalyzes the production of oxygen radicals, known as the Fenton reaction. Therefore, the uptake of Fe, storage mechanisms, and Fe cofactor biosynthesis must be tightly regulated (8). In their natural environment, plants are generally Fe starved, and the Fe deficiency response is engaged to maximize uptake. However, when Fe becomes available through new root growth or changes in the environment, uptake needs to be switched off immediately to avoid Fe overload. In addition to internalization of IRT1 (9–11), the activity of upstream transcription factors is down-regulated. FIT protein levels are controlled by 26S-proteasome-dependent turnover, which, paradoxically, is initiated during Fe deficiency while FIT is transcriptionally up-regulated (12, 13). It has been proposed that a short-lived pool of FIT is important to anticipate a sudden increase in Fe in the environment. However, the E3 ligase(s) that may facilitate the turnover of FIT have so far not been identified.

**Fig. 1.**
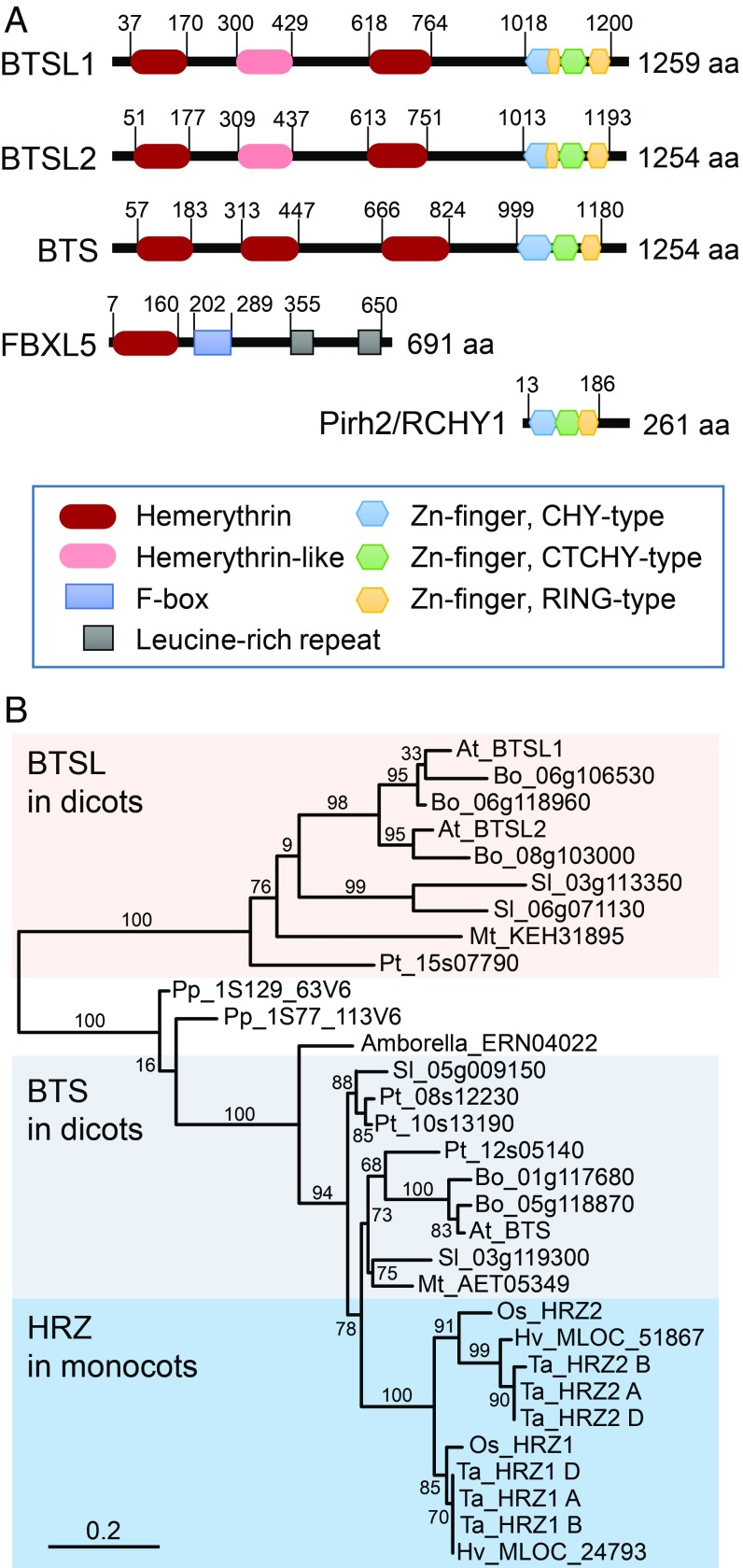
BTSL are uniquely found in dicotyledonous plants. (*A*) Domain organization of the BTSL and BTS proteins and closest mammalian homologs. (*B*) Phylogenetic tree of BTS homologs from selected plant species. Sequences were found by BLASTing the amino acid sequences of *Arabidopsis* BTS, BTSL1, BTSL2, and rice HRZ1 in Ensembl Plants (http://plants.ensembl.org). Species used: Amborella, *Amborella trichocarpa*; At, *A. thaliana*; Bo, *Brassica oleracea*; Hv, *Hordeum vulgare*; Mt, *Medicago truncatula*; Os, *Oryza sativa*; Pp, *Physcomitrella patens*; Pt, *Populus trichocarpa*; Sl, *Solanum lycopersicum*; Ta, *Triticum aestivum*. Numbers next to branches indicate bootstrapping values for 100 replications. The scale bar indicates the rate of evolutionary change expressed as number of amino acid substitutions per site.

Among the 20 different E3 ligases that are up-regulated during Fe deficiency (4, 14, 15) is a small gene family encoding E3 ubiquitin ligases with N-terminal hemerythrin motifs. These include BRUTUS (BTS) in *Arabidopsis* (16, 17); and the hemerythrin motif-containing RING- and zinc (Zn)-finger proteins (HRZ1, HRZ2) in rice (18). The BTS and HRZ proteins have been characterized as negative regulators of Fe homeostasis, since *bts* and *hrz* mutant lines accumulated Fe and exhibited increased tolerance to Fe deficiency. The combination of an Fe-binding hemerythrin motif associated with an E3 ligase is also found in mammalian FBXL5 (F-box/LRR protein 5, Fig. 1*A*). The stability of FBXL5 is regulated by Fe and oxygen, providing a switch to promote the ubiquitination and degradation of IRP1 and IRP2, iron regulatory proteins 1 and 2 (19–21). However, there are no functional homologs of IRP1 and IRP2 in plants; therefore, the BTS/HRZ proteins must have different targets. Their E3 ligase domain shows striking homology to the mammalian Pirh2/RCHY1 E3 ligase (ref. 22 and Fig. 1*A*), which regulates the levels of p53 transcription factor (23, 24). In *Arabidopsis*, BTS was shown to interact with, and affect the stability of, the transcription factors bHLH105 (ILR3) and bHLH115, which are involved in Fe signaling (17). In rice, HRZ1 targets PRI1, the homolog of ILR3, for degradation (25). Two genes homologous to *BTS* are found in the *Arabidopsis* genome, named *BRUTUS-LIKE1* (*AT1G74770*) and *BRUTUS-LIKE2* (*AT1G18910*). The *btsl1 btsl2* double knockout mutant accumulated Fe and was more tolerant to Fe deficiency (26). A triple mutant of *btsl1*, *btsl2,* and *bts* had an enhanced phenotype; therefore, it was suggested that the function of *BTSLs* is redundant with *BTS*.

Here we show that the function of the BTSL proteins differs from BTS in their tissue-specific expression and ubiquitination targets. *BTSL1* and *BTSL2* are expressed predominantly in the root epidermis and cortex and are coregulated with Fe uptake genes, whereas *BTS* is coexpressed with other Fe homeostasis genes. On standard medium, *btsl1 btsl2* double mutants accumulated moderate amounts of Fe, but when challenged with Fe resupply after a period of deficiency, large amounts of Fe accumulated as the mutant plants failed to rapidly switch off the transcription of *FRO2* and *IRT1*. Using a range of assays, we show that the E3 ligase domains of BTSL1 and BTSL2 were able to ubiquitinate FIT in vitro and target the transcription factor for degradation.

## Results

### BTSL1 and BTSL2 Proteins Are Unique to Dicotyledonous Plants.

To study the evolutionary relationship between hemerythrin E3 ligases in plants, we performed a phylogenetic analysis of homologous protein sequences across the plant kingdom. Interestingly, *Arabidopsis* BTSL1 and BTSL2 are in a separate clade from BTS, and the BTSL clade is unique to dicotyledonous species (Fig. 1*B*).

In terms of domain organization, the *Arabidopsis* BTSL1/2 proteins have 2 predicted hemerythrin motifs compared to 3 motifs in BTS, and a C-terminal CHY/RING Zn-finger domain (Fig. 1*A*). The 2 hemerythrin motifs of BTSL1/2 correspond to the first and third motif in BTS. Further inspection revealed a degenerate hemerythrin sequence in BTSL1/2, not recognized by motif searching algorithms, corresponding to the second hemerythrin motif of BTS. Although the 4 conserved histidine residues found in canonical hemerythrin proteins are lacking, the sequence is predicted to form a 4 α-helical bundle typical of hemerythrins (*SI Appendix*, Fig. S1).

The C-terminal Zn-finger domain has 80% amino acid identity between BTSL1 and BTSL2, and 65% identity with BTS. This particular type of CHY/RING Zn-finger domain is also found in 4 other *Arabidopsis* E3 ligases such as MIEL1 (27), and in 1 mammalian E3 ligase, Pirh2/RCHY1 (23). Structural studies of Pirh2 using NMR showed a unique arrangement of Zn-fingers, with a total of 9 Zn-binding sites. The first 2 Zn-fingers (CHY- and CTCHY-type) form a “tweezer” together with the C-terminal amino acids for interaction with its target, the transcription factor p53. The RING Zn-finger domain provides the interaction platform for the E2 ligase (24). It is interesting to note that BTSL proteins have an extra RING Zn-finger motif inserted between the CHY and CTHY Zn-finger motifs (Fig. 1*A*).

### *BTSL1* and *BTSL2* Promoters Are Activated by Fe Deficiency in Specific Root Tissues.

**Fig. 2.**
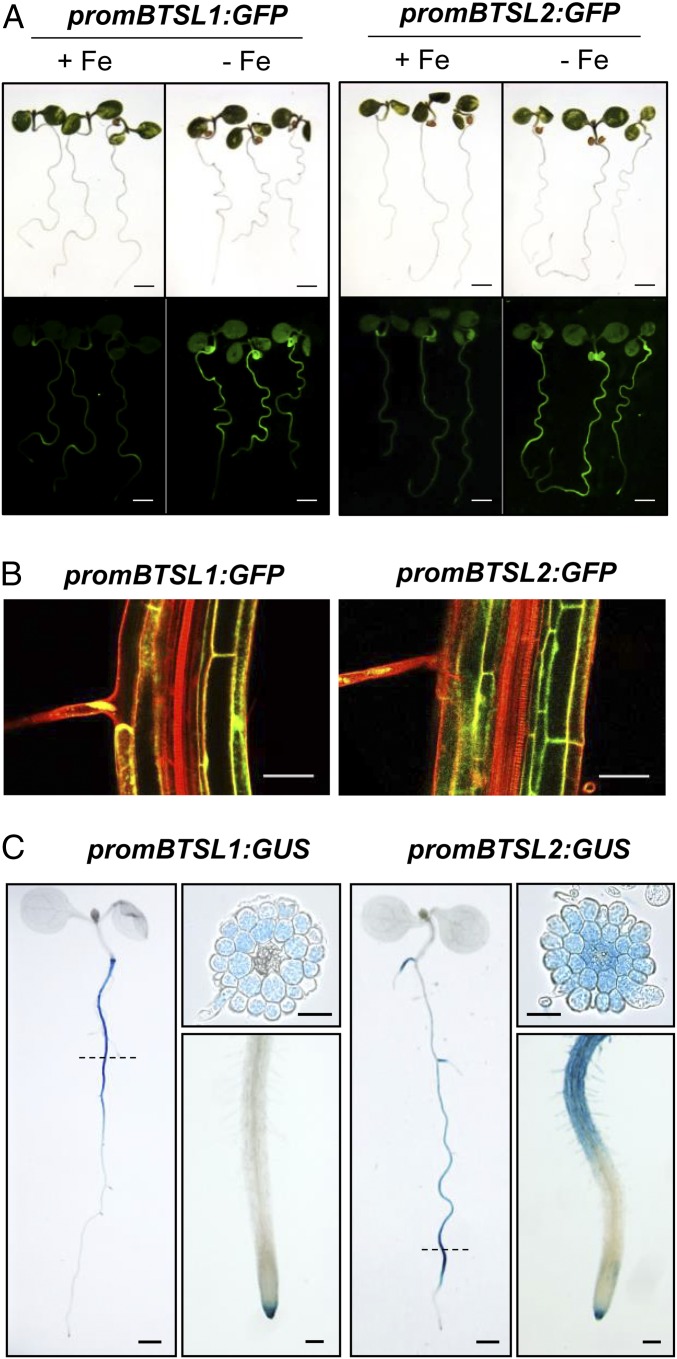
Promoter activity of *BTSL1* and *BTSL2*. Promoter sequences of *BTSL1* (−880 nt) and *BTSL2* (−2097 nt) were inserted upstream of *eGFP-GUS* and the constructs were stably expressed in wild-type *Arabidopsis*. (*A*) GFP fluorescence in seedlings grown on medium with 50 μM FeEDTA (+Fe) or with 100 µM ferrozine to deplete Fe (−Fe) for 6 d. (Scale bar, 1 mm.) (*B*) GFP fluorescence in optical transverse sections of roots in 6-d-old seedlings grown −Fe. Cell walls were stained with propidium iodide (red). (Scale bar, 50 μm.) (*C*) GUS activity staining in seedlings grown −Fe for 8 d. Scale bar is 0.5 mm for whole seedlings, 100 μm for a close-up of the root tip, and 50 μm for root cross-sections. Images are representative of 3 independent lines for each promoter construct.

*BTSL1* and *BTSL2* are expressed predominantly in roots under Fe deficiency (26, 28). To investigate where in the roots and in which cell types *BTSL1* and *BTSL2* are expressed, the promoter regions of these genes were cloned upstream of an *eGFP-GUS* reporter gene (29). Seeds were germinated on agar plates with minimum salts (*SI Appendix*, *Materials and Methods*) supplemented either with 50 µM FeEDTA (+Fe) or with 100 µM ferrozine to effectively deplete Fe (−Fe). After 5 d of growth, GFP was highly induced in roots −Fe, whereas no GFP fluorescence was observed +Fe, except for auto-fluorescence in the seed coat (Fig. 2*A*). In older seedlings which had developed lateral roots, the expression pattern of the *BTSL1* and *BTSL2* promoters started to differentiate from each other: GUS staining showed that *BTSL1* is expressed in the upper half of the root under Fe deficiency, whereas *BTSL2* is expressed in the lower half of the root, predominantly in the differentiation zone with root hairs (Fig. 2*C*). *BTSL1* and *BTSL2* promoter-GUS activity was also found in the root cap (columella), but not in the root meristem or elongation zone. Cross-sections of the GUS-stained roots (Fig. 2*C*) and imaging of GFP expression in the longitudinal plane (Fig. 2*B*) showed that both promoters are induced in the epidermis, including root hairs, and cortex cells. *BTSL2* was also expressed in the endodermis and stele in the differentiation zone (Fig. 2 *B* and *C*). The expression patterns of *BTSL1* and *BTSL2* contrast with the pattern observed for *BTS*, which has little promoter activity in the epidermis and cortex, but was strongly up-regulated in the stele in response to Fe deficiency (17). Moreover, *BTS* is expressed in leaves and in embryos (17) which lack expression of *BTSL1* and *BTSL2* (*SI Appendix*, Fig. S2).

Using publicly available microarray data, we built coexpression networks around *BTSL1, BTSL2, and BTS*, then filtered for Fe-regulated genes (30). First, we found that *BTSL1* and *BTSL2* are coregulated with each other but not with *BTS* (*SI Appendix*, Fig. S3 and Table S1). Second, we noticed that a large network could be built around *BTSL1* and *BTSL2* using microarray data from root samples, but correlations with *BTS* were only found when using data sets from shoots. *BTSL1* and *BTSL2* are coregulated with the Fe transporter gene *IRT1* and genes for the biosynthesis and export of coumarin-derived Fe chelators (*4CL2, F6’H1* and *PDR9*). Also coexpressed with *BTSL1* and *BTSL2* are the transcription factor *FIT* and its partner *bHLH39*. Basically, the entire root Fe uptake regulon is found, except for *bHLH38* and *FRO2*, but these genes are not present on the microarray chip that is most commonly used. The *BTS* network includes the transcription factor *POPEYE* (*PYE*), as previously documented (16), and the ferric reductase *FRO3* (*SI Appendix*, Fig. S3*B* and Table S1). The different coexpression networks of *BTSL1/2* and *BTS* correlate well with their tissue-specific expression patterns.

### BTSL1 and BTSL2 Prevent Excess Fe Uptake under Fluctuating Levels.

To further compare the functions of BTSL1, BTSL2, and BTS, we obtained T-DNA insertion lines. Three independent mutant lines were selected for *BTSL1* (*btsl1-1, btsl1-2, and btsl1-3*), but only 1 T-DNA insertion line was available for *BTSL2* (*btsl2-2*) (*SI Appendix*, Fig. S4*A*). The *btsl1-1* allele has been described as *btsl1* in ref. 26. The expression of *BTSL1* was virtually abolished in the *btsl1-1* and *btsl1-3* alleles, but residual expression remained in *btsl1-2* (*SI Appendix*, Fig. S4*B*). The *btsl2-2* mutant lacked detectable levels of *BTSL2* transcript, while *BTSL1* expression was ∼3-fold higher than in wild type −Fe. A double knockout line was produced by crossing *btsl1-1* with the *btsl2-2* line. The *btsl1-1 btsl2-2* double mutant was genetically complemented by either *BTSL1:YFP* or *BTSL2:GFP* (see below).

Mutant lines were tested for phenotypes on a range of Fe concentrations, from 0 to 500 µM FeEDTA (*SI Appendix*, Fig. S5*A*). Single insertion lines showed no obvious growth phenotype as previously reported (26). On the other hand, the *btsl1-1 btsl2-2* double mutant line showed subtle but noticeable phenotypes in response to both Fe deficiency and Fe excess. After 4 d without Fe, the *btsl* double mutant appeared less chlorotic than wild type, which was confirmed by chlorophyll measurements (*SI Appendix*, Fig. S5 *A* and *B*). The *btsl2-2* mutant allele also retained some chlorophyll, suggesting that *BTSL1* and *BTSL2* are not fully redundant. In the presence of excess Fe, growth of the *btsl* double mutant was significantly impaired compared to wild type or the single mutants, associated with a 2-fold accumulation of Fe in the shoots (*SI Appendix*, Fig. S5 *A* and *C*).

**Fig. 3.**
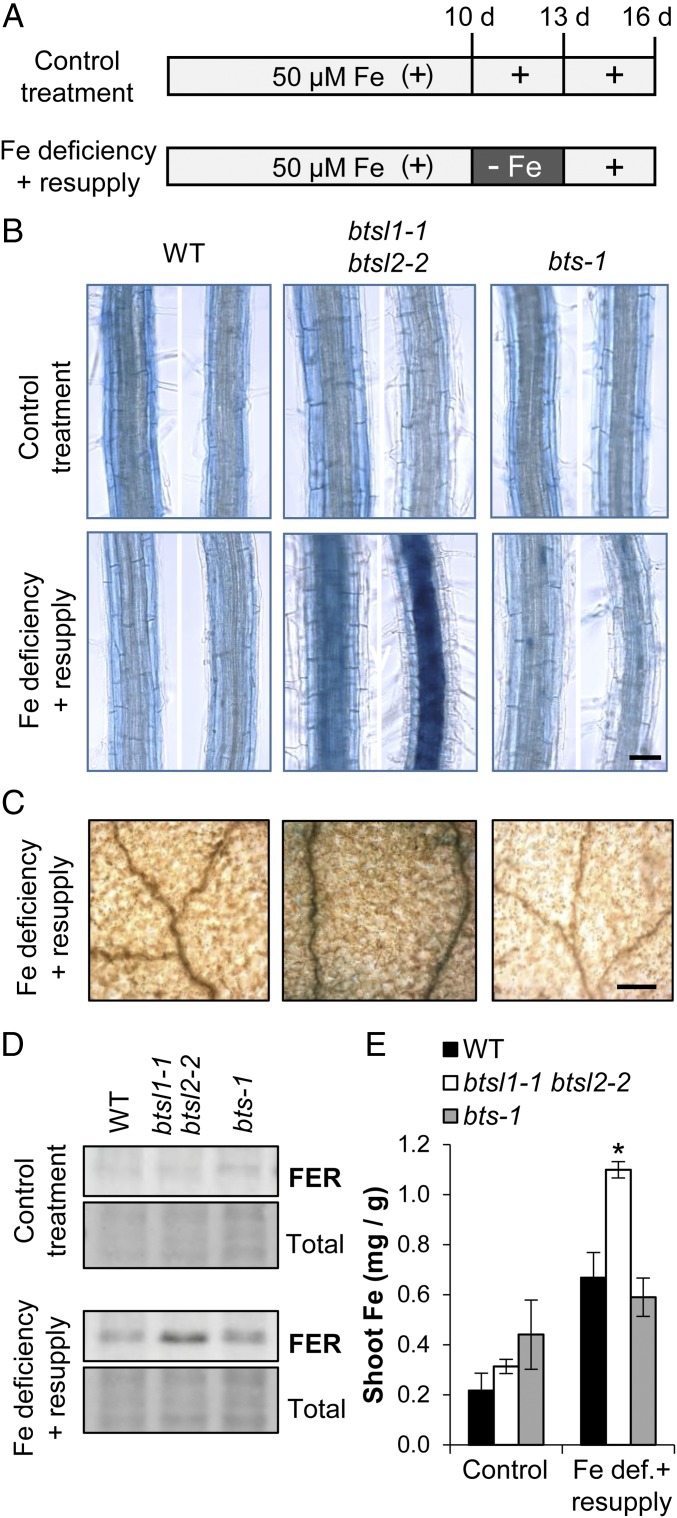
The *btsl* double mutant hyper-accumulates Fe after a period of Fe deficiency. (*A*) Diagram of the Fe treatments used in this study. Seedlings were germinated on medium with 50 μM FeEDTA. On day 10, seedlings were transferred to a new plate (control treatment) or to medium depleted of Fe (100 μM ferrozine). After 3 d, seedlings were transferred back to medium with 50 μM FeEDTA for another 3 d. (*B*) Perls’ Prussian Blue staining for Fe in roots in wild type, *btsl* double mutant and *bts-1* seedlings following control (*Top*) and Fe deficiency-resupply treatments (*Bottom*) according to the diagram in *A*. The images show a section of the differentiation zone. (Scale bar, 50 μm.) (*C*) Perls’ Prussian Blue staining of leaves after the Fe deficiency-resupply treatment. The images are a close-up of the adaxial leaf surface with veins. (Scale bar, 0.2 mm.) (*D*) Immunoblot of ferritin (FER) protein in roots and (*E*) Fe concentrations in shoots of wild type, *btsl* double mutant, and *bts-1* seedlings after control and Fe deficiency-resupply treatments. Bars represent the mean of 3 biological replicates of 10 pooled plants each ±SD (*P < 0.05 using a 2-tailed *t* test).

We tested whether the *BTSL* genes have a specific function in the transition from Fe deficiency to Fe sufficiency. Wild-type seedlings and *btsl* double mutants were grown up with 50 μM FeEDTA (+Fe) for 10 d and then transferred to −Fe medium for 3 d to induce Fe deficiency. After that, plants were resupplied with Fe and sampled after 3 d (Fig. 3*A*). While there were no obvious growth phenotypes, the *btsl* double mutant accumulated large amounts of Fe in the central cylinder of the root and in leaf veins as shown by Perls’ staining (Fig. 3 *B* and *C*). Ferritin as a marker for Fe status accumulated in the roots of the *btsl* double mutant (Fig. 3*D*), and the shoots contained 4-fold more Fe (Fig. 3*E*). Iron did not accumulate when *btsl* double mutants were grown continuously on 50 μM Fe. For comparison, we also grew the viable *brutus-1* (*bts-1*) mutant (17) under the same conditions. The *bts-1* behaved as wild type and did not show increased Perls’ staining in any part of the root or leaves after the Fe deficiency-resupply treatment. Interestingly, we found that wild-type plants, when subjected to deficiency and resupply, contained 3-fold more Fe compared to continuous growth on 50 µM Fe (Fig. 3*E*). It is likely that up-regulation of the Fe uptake machinery during Fe deficiency leads to a sudden influx of Fe when this is resupplied, but to levels that can be controlled by redistribution in wild-type plants.

In summary, our data show that the *btsl* double mutant is unable to limit Fe uptake when this is supplied after a period of Fe deficiency. Moreover, lack of Fe accumulation in the *bts-1* mutant under these conditions suggest that BTSLs and BTS have different functions.

### Transcriptional Down-Regulation of Fe Uptake Genes Is Delayed in the *btsl1-1 btsl2-2* Double Mutant.

**Fig. 4.**
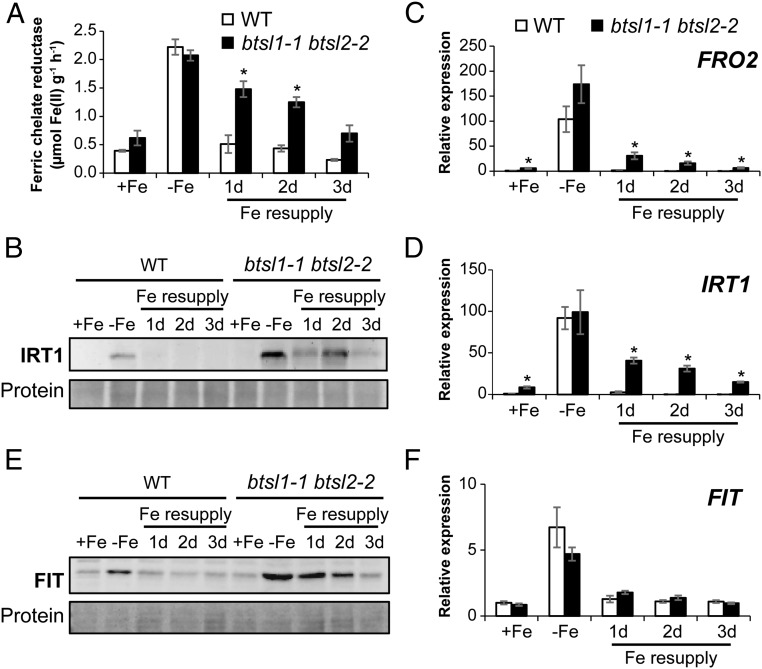
The *btsl* double mutant has delayed down-regulation of FRO2, IRT1, and FIT upon Fe resupply. (*A*) Ferric chelate reductase activity as a measure for FRO2 protein activity in roots of wild type and *btsl1-1 btsl2-2* double mutants. Bars represent the mean of 3 biological replicates (*n* = 5 seedlings/assay) ± SE (*P < 0.05 using a 2-tailed *t* test). (*B*) Immunoblot of IRT1 protein levels. (*C*, *D*, and *F*) Expression of *FRO2, IRT1, and FIT,* respectively, determined by quantitative RT-qPCR in roots of wild type and *btsl1-1 btsl2-2* mutants. All values are relative to wild type +Fe and the mean of 3 biological replicates ±SE (*P < 0.05 using a 2-tailed *t* test). (*E*) Immunoblot of FIT protein. Quantification of 3 independent immunoblots can be found in *SI Appendix*, Fig. S7*A*.

Next, we investigated the levels of FRO2 and IRT1, 2 key players in Fe uptake, during Fe deficiency and resupply. Wild-type seedlings showed a ∼6-fold induction in FRO2 activity in response to Fe deficiency, and down to basal levels 1 d after Fe resupply (Fig. 4*A*). In the *btsl* double mutant, FRO2 enzyme activity remained high after Fe resupply: the activity was 4-fold induced after 1 d, 3-fold after 2 d, and it was still nearly double the basal wild-type levels after 3 d (Fig. 4*A*). Of the single mutant lines, *btsl1-1* behaved like wild type, but *btsl2-2* showed a slight delay in switching off FRO2 activity (*SI Appendix*, Fig. S6*A*). The sustained induction of FRO2 activity in the *btsl* double mutant was restored by expressing either *35S:BTSL1-YFP* or *35S:BTSL2-GFP* (*SI Appendix*, Fig. S6*B*).

IRT1 protein levels in wild-type plants were strongly increased under Fe deficiency, and down after Fe resupply. In the *btsl* double mutant, IRT1 was not detected under standard conditions (+Fe), but, after 3 d of Fe deficiency, was present at much higher levels than in wild-type roots. Upon Fe resupply, the IRT1 protein levels remained high (Fig. 4*B*). The sustained presence of FRO2 and IRT1 would explain the increased Fe accumulation in the *btsl* double mutant following the Fe deficiency-resupply treatment.

To investigate which step of FRO2 and IRT1 expression is misregulated, we analyzed transcript levels of *FRO2* and *IRT1* by RT-qPCR. Interestingly, under control conditions (+Fe), *FRO2* and *IRT1* transcript levels were already increased 5- to 8-fold, respectively, in the *btsl* double mutant compared to wild type (Fig. 4 *C* and *D*). After 3 d of Fe deficiency, transcription of *FRO2* and *IRT1* was strongly up-regulated by a similar magnitude in both mutant and in wild type. However, upon Fe resupply, *FRO2* and *IRT1* transcript levels remained high in the *btsl* double mutant (Fig. 4 *C* and *D*), matching the sustained FRO2 activity and IRT1 protein levels, respectively.

The transcription of *FRO2* and *IRT1* is directly regulated by FIT together with 1 of 4 partially redundant bHLH transcription factors, bHLH38, bHLH39, bHLH100, or bHLH101 (6, 7). To investigate the levels of FIT protein, specific antibodies were raised against recombinantly expressed protein. FIT has a predicted molecular weight of 35.5 kDa, but on SDS/PAGE it migrates at 55 kDa, as previously shown (12). In wild-type seedlings, FIT was increased under Fe deficiency and decreased after resupply, as expected (Fig. 4*E* and *SI Appendix*, Fig. S7). In the *btsl* double mutant –Fe, the levels of FIT protein were 2-fold higher than in wild type, and only slowly diminished after Fe resupply. Strikingly, there was no significant change in *FIT* transcript levels in *btsl1 btsl2* compared to wild type in any of the tested Fe conditions (Fig. 4*F*). These findings indicate that FIT protein synthesis or turnover are misregulated in the *btsl* mutant. In contrast, the levels of FIT protein in the *bts-1* mutant resemble those of wild type, and *FRO2* and *IRT1* expression are normal under changing Fe conditions (*SI Appendix*, Fig. S7). Thus, it is likely that BTSL and BTS differ in their downstream targets.

### The E3 Ligase Domains of BTSL1 and BTSL2 Interact with FIT.

**Fig. 5.**
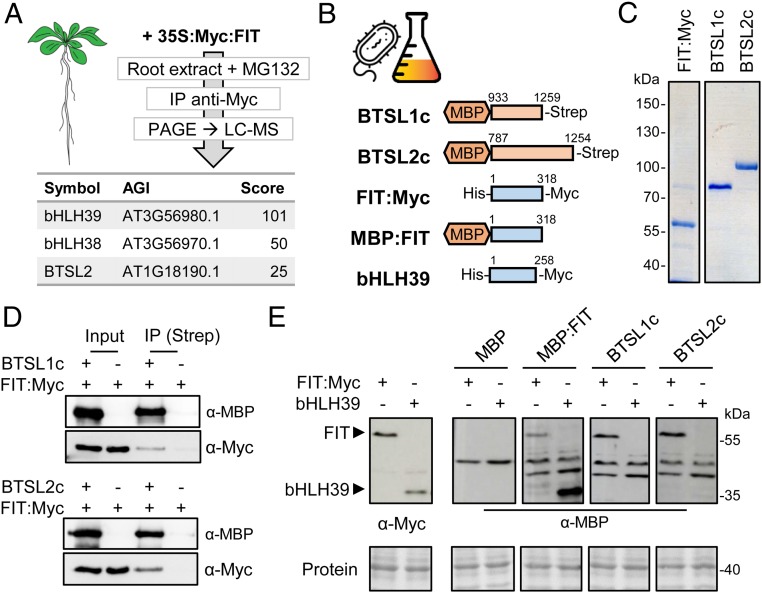
The C-terminal domain of BTSL1 or BTSL2 interacts with FIT. (*A*) BTSL2 coimmunoprecipitated (IP) with FIT from extracts of *Arabidopsis* roots. Proteins were separated by SDS/PAGE and identified by LC-MS. (*B*) Diagram of the recombinant proteins produced in *E. coli* for in vitro analyses. (*C*) Purified FIT, BTSL1c, and BTSL2c proteins stained with Coomassie blue. (*D*) Interaction between the C-terminal E3 ligase domain of BTSL proteins and FIT shown by immunoprecipitation. (*E*) Far-Western blot analysis to compare the interaction of BTSL proteins with FIT and its partner bHLH39. Protein extracts of bacteria producing FIT:Myc or bHLH39:Myc were separated by SDS/PAGE, blotted, and incubated with the indicated proteins, followed by immunodetection of MBP. Ponceau staining shows equal protein loading.

To investigate if FIT is a direct target of the BTSL E3 ubiquitin ligases, we tested for protein interaction. Because BTSL protein levels are very low even when expressed using the 35S promoter (*SI Appendix*, Fig. S8), we used FIT as a bait to pull down interacting proteins. Plants expressing *35S:Myc-FIT* were grown and roots were harvested for protein extraction in the presence of the proteasomal inhibitor MG132. Proteins bound to Myc affinity resin were separated by SDS/PAGE gel, followed by mass spectrometry for identification. Among the proteins pulled down with Myc-FIT were bHLH38 and bHLH39 (Fig. 5*A*), which are known to form stable heterodimers with FIT for transcriptional activity (6). In addition, we detected peptides matching BTSL2 (*P* < 0.05) in pull-downs from Myc-FIT expressing plants, but not in control plants (Fig. 5*A*). Confirmation of the interaction by yeast 2-hybrid assays was hampered by auto-activation. As an alternative approach, protein interactions were validated in vitro using recombinant proteins expressed in *Escherichia coli*. The C-terminal half of BTSL1 (residues 933–1259) and BTSL2 (residues 787–1254), encompassing the predicted binding sites for E2 protein and putative target proteins, were fused to maltose-binding protein (MBP) at the N terminus to improve protein stability (Fig. 5*B*). A C-terminal Strep-tag was added to the BTSLc proteins for affinity purification. FIT was expressed with His and Myc-tags at the N- and C-termini, respectively (Fig. 5*B*). The FIT and BTSLc proteins were purified by affinity resins and the purity of the recombinant proteins was verified by SDS/PAGE and Coomassie staining (Fig. 5*C*). FIT and BTSLc proteins were mixed and BTSL1c or BTSL2c were recaptured using Strep-tactin resin. FIT was also pulled down, but not when BTSL was omitted (Fig. 5*D*).

It is possible that the BTSL proteins primarily interact with bHLH38 and bHLH39, which would result in coimmunoprecipitation of FIT. To test this, bHLH39 was expressed in *E. coli*, but because of poor protein stability, far-Western blot analysis was used to probe the interaction with BTSL E3 ligase domains. Immunodetection of the Myc-tag confirmed the expression of FIT and bHLH39 (Fig. 5 *E*, *Left*). Purified MBP, MBP:FIT or MBP:BTSLc were incubated with the membrane, followed by antibody detection of MBP. MBP:FIT bound strongly to bHLH39 and weakly to itself, as expected. The immuno-signals were clearly separated from an aspecific band in the MBP control. Both MBP:BTSL1c and MBP:BTSL2c bound to FIT, but not to bHLH39 (Fig. 5*E*). Thus, bHLH39 is unlikely to be a target of BTSL1 or BTSL2, in agreement with RT-qPCR analysis showing that misregulation of bHLH39 is at the transcriptional level (*SI Appendix*, Fig. S9).

### The BTSL Proteins Display E3 Ligase Activity and Promote Degradation of FIT.

**Fig. 6.**
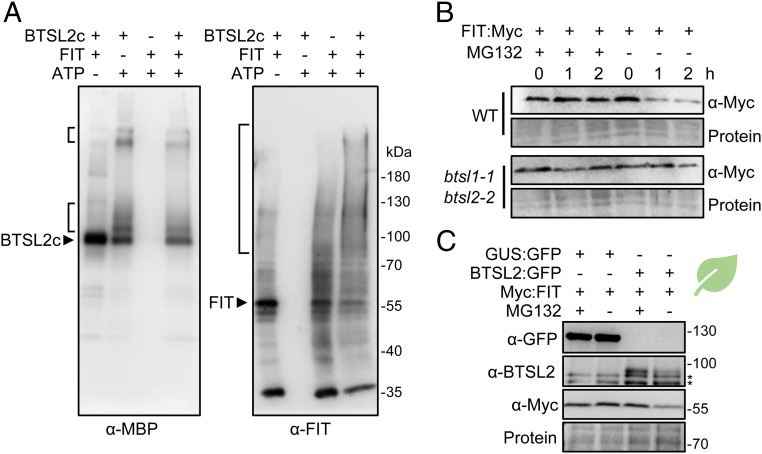
The C-terminal domain of BTSL2 catalyzes FIT ubiquitination and degradation. (*A*) In vitro ubiquitination activity of MBP:BTSL2c in the presence of [E1 + E2] and ATP. Proteins were separated by SDS/PAGE and higher molecular weight protein products were detected by Western blot analysis using anti-MBP antibodies for MBP:BTSL2c and purified anti-FIT antibodies for FIT. (*B*) Cell-free degradation of recombinant FIT in *Arabidopsis* root extracts. Protein extracts from 10-d-old wild-type (WT) and *btsl* double mutant seedlings were spiked with recombinant FIT:Myc protein and incubated in the presence of either MG132 (proteasome inhibitor) or DMSO (solvent) for the indicated length of time. (*C*) Steady state protein levels present in extracts from *N. benthamiana* leaves transiently expressing Myc:FIT and BTSL2c:GFP or GUS:GFP in the presence or absence of MG132. BTSL2c:GFP could be detected with anti-BTSL2c antibodies but not with commercial anti-GFP. Aspecific bands are marked (*). Ponceau staining shows equal loading.

To test if the BTSL proteins are functional E3 ligases, purified MBP:BTSL1c or MBP:BTSL2c were incubated with E1 ubiquitin-activating enzyme, E2 ubiquitin-conjugating enzyme (UbcH5c), ubiquitin, and ATP. Immunoblot analysis using antibodies against MBP showed additional higher molecular weight forms of BTSL1c and BTSL2c when ATP was present (Fig. 6 *A*, *Left* and *SI Appendix*, Fig. S10). These are likely to be poly-ubiquitinated forms, showing that the E3 ligase domain has self-ubiquitination activity, which is commonly observed for these enzymes (31). Next, FIT was added to the reaction mixture as a potential substrate of BTSL2, which is more active than BTSL1 (*SI Appendix*, Fig. S10) and therefore used in subsequent experiments. Immunodetection of FIT revealed higher molecular weight forms typical of ubiquitinated protein products in the presence of BTSL2 but not in control reactions (Fig. 6 *A*, *Right*).

To test if BTSL2 is able to promote the proteasomal degradation of FIT in vivo we performed cell-free degradation assays. First, protein extracts from wild-type *Arabidopsis* seedlings and the *btsl* double mutant were incubated with recombinant FIT protein. In the absence of the proteasomal inhibitor MG132, FIT protein levels were decreased within 1 h of incubation in wild-type extracts, but not in extracts from *btsl* double mutants (Fig. 6*B*). The cell-free degradation assay was also performed using *Nicotiana benthamiana* leaves transiently expressing Myc:FIT and BTSL2c:GFP and showed similar results (*SI Appendix*, Fig. S10). To further show support for the BTSL-dependent degradation of FIT, the proteins were expressed in *N. benthamiana* leaves followed by Western blot detection. In the absence of MG132, Myc:FIT protein levels were diminished when BTSL2c:GFP was coexpressed, but not when GUS:GFP was coexpressed (Fig. 6*C*). These results confirm that BTSL2 is implicated in FIT proteasomal degradation. Interestingly, BTSL2c:GFP protein was also stabilized by MG132, highlighting again its self-ubiquitination activity and tight regulation of its abundance, which is extremely low, even when expressed using strong constitutive promoters.

Collectively, our data show that the E3 ligase domain of either BTSL1 or BTSL2 can interact with FIT, and that BTSL2 is able to poly-ubiquitinate FIT in vitro and promote the proteasomal degradation of this transcription factor.

## Discussion

Despite substantial differences in Fe homeostasis mechanisms, plants and animals employ hemerythrin E3 ubiquitin ligases for posttranslational regulation of Fe uptake. Functional comparison of the hemerythrin E3 ligases in *Arabidopsis* showed that the *BTSL1* and *BTSL2* genes have a distinct function from *BTS*, and are not redundant paralogs as previously proposed (26). *BTSL* genes have evolved specifically in dicotyledonous plants (Fig. 1*B*) and are coregulated with the Fe uptake machinery in the root (*SI Appendix*, Fig. S3). The different expression patterns of BTSL1/2 and BTS (Fig. 2 and *SI Appendix*, Fig. S2) suggest that the E3 ligases target different proteins for degradation. Indeed, we found that the partially redundant BTSL1 and BTSL2 target the bHLH transcription factor FIT for degradation (Figs. 4–6), whereas BTS has previously been found to interact with and induce the degradation of the bHLH proteins ILR3 and bHLH115 (17). In rice, mutant lines of *HRZ1* and *HRZ2* showed misregulation of Fe-regulated transcripts including *OsIRO2* and *OsIRO3* (18). More recently, it was shown that HRZ1 poly-ubiquitinates OsPRI1, the homolog of ILR3 and a transcriptional regulator of *OsIRO2* (25). The target of HRZ2 has not yet been identified.

*Arabidopsis BTSL1* and *BTSL*2 are closely related in sequence and, based on phylogeny, result from an ancient chromosome segment duplication (Fig. 1*B*). Although mutant analysis showed that the function of the 2 genes is overlapping, *BTSL2* appears to be the dominant paralog. Comparing full knockout alleles of *BTSL1* and *BTSL2*, the *btsl2-2* mutant has a weak phenotype on its own, including residual chlorophyll in Fe-deficient seedlings (*SI Appendix*, Fig. S5). These phenotypic observations are in agreement with residual FRO2 activity in *btsl2-2*, but not *btsl1-1*, upon Fe resupply (*SI Appendix*, Fig. S6). Of the 2 *BTSL* genes, the expression pattern of *BTSL2* is more similar to that of *FIT*, with both transcripts being produced in the differentiation zone with root hairs where nutrients are taken up (Fig. 2) (4, 32). Either *BTSL1* is slowly degenerating and becoming obsolete, or it is taking on a different function, with different ubiquitination targets. For both BTSL1 and BTSL2, it will be interesting to explore whether they have other targets besides FIT. In particular, the misregulation of *bHLH38* and *bHLH39* transcripts in the *btsl* double mutant (*SI Appendix*, Fig. S9) may be explained by this possibility if 1 of the BTSLs were to target ILR3 for degradation (33).

**Fig. 7.**
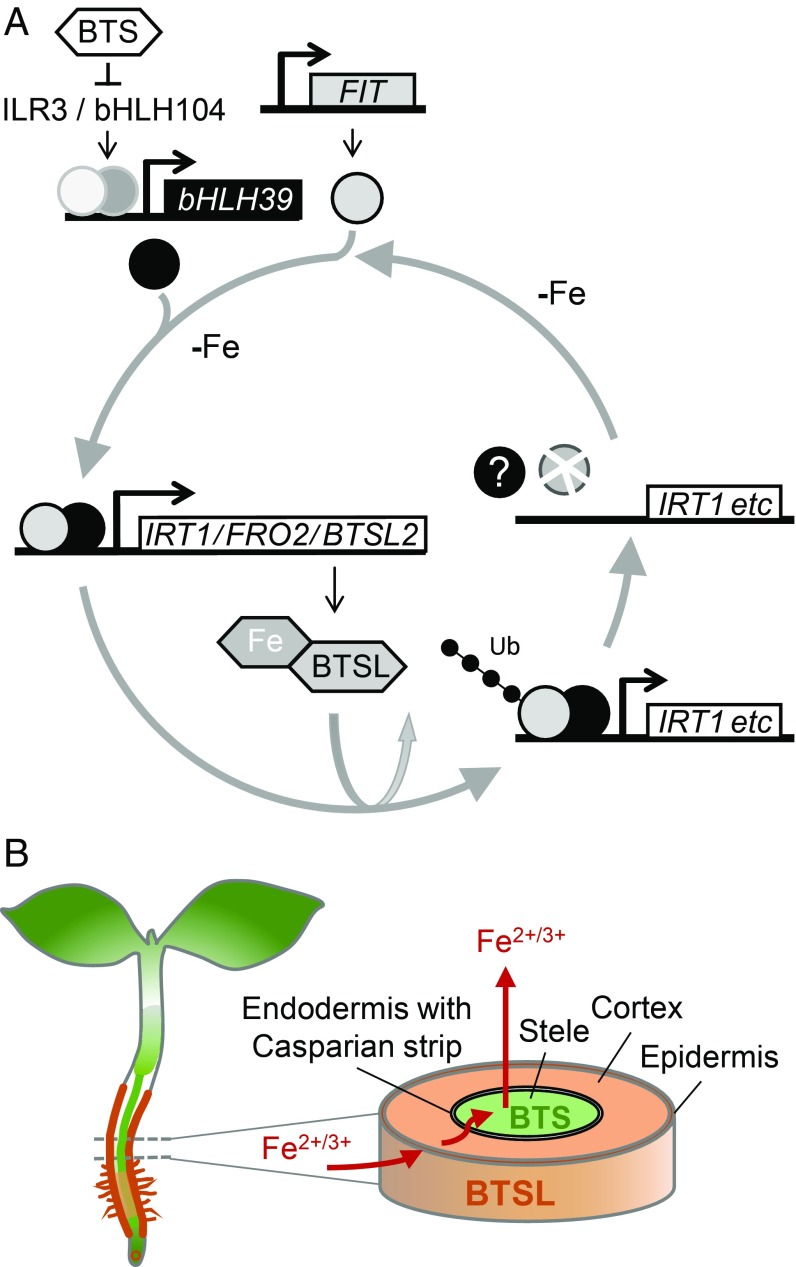
Proposed modes of action of BTSL and BTS in Fe homeostasis. (*A*) The transcription of *FIT* is up-regulated under Fe deficiency (top), resulting in increased levels of FIT protein, which forms a dimer with bHLH39 (or bHLH38). The FIT + bHLH39 dimer up-regulates the transcription of genes for Fe uptake, including *IRT1* and *FRO2*. The transcript levels of *BTSL2* are also controlled by FIT. The BTSL proteins act as E3 ubiquitin ligases and promote the degradation of FIT. Iron binding to the N-terminal hemerythrin domains of BTSL is likely to affect their stability and forms another layer of regulation. The constant turnover of FIT is proposed to facilitate rapid down-regulation of the Fe deficiency response when Fe becomes available. (*B*) *BTSL1* and *BTSL2* (orange) are expressed primarily in the epidermis and cortex cells, where they regulate the level of FIT protein. BTS (green) is predominantly expressed in the stele and shoot, and regulates the levels of ILR3. Thus, BTSL proteins provide a first protection mechanism against Fe overload outside the endodermis and Casparian strip, whereas BTS protects against Fe overload inside this barrier for nutrients.

Based on our results showing BTSL-dependent modification of FIT in vitro and previous studies on FIT protein dynamics (12), we propose that BTSLs are required for constant recycling of FIT, which helps to rapidly switch off its transcriptional activity when Fe is resupplied (Fig. 7*A*). A parallel can be drawn with the role of RNF in mouse, an E3 ligase that turns over the transcription factor STAT1 for instant down-regulation of the inflammation response (34). In Fe-deficient plants, inhibition of the 26S-proteasome by MG132 resulted in a lower transcriptional activity of FIT under Fe deficiency (12), presumably because promoters are populated with ubiquitinated, inactive FIT which cannot be removed. In contrast, there was no difference in transcriptional activity of FIT under Fe deficiency in the *btsl* double mutant compared to wild type (Fig. 4*A*), which could be explained by FIT remaining active when not ubiquitinated in the *btsl* mutant.

Because *BTSL2* is under transcriptional control by FIT (4, 32), a negative feedback loop operates continuously to regulate FIT protein levels (Fig. 7*A*). Another layer of regulation is likely to be provided by Fe binding to the N-terminal hemerythrin domain of BTSL proteins, similar to BTS/HRZ and the mammalian FBXL5 protein (35). Whereas FBXL5 protein is stabilized by Fe binding (19, 20), the *Arabidopsis* BTS protein appears to be destabilized by Fe (17). The effect of Fe on BTSL protein stability remains to be investigated. Furthermore, our results suggest that the heterodimeric partner protein bHLH39 is not targeted by BTSLs. Indeed, targeting FIT rather than bHLH38/39/100/101 would be more effective, as FIT is nonredundant, and many signaling pathways converge on this protein to regulate Fe homeostasis (36).

A closer look at the expression patterns of the *BTSL1*, *BTSL2,* and *BTS* genes suggests a demarcation by the endodermis: the 2 *BTSL* genes are predominantly expressed in the root epidermis and cortex, whereas *BTS* is expressed in the root stele and in the shoot. The endodermal Casparian strip, when made impermeable by suberin, is well known to affect metal and nutrient distribution, providing an effective barrier against nutrient overload in the leaves (37, 38). We propose a model where BTSL proteins act as a primary defense mechanism against excess Fe uptake in the root. A second defense mechanism against Fe overload is then regulated by the BTS protein in the stele and leaf tissues, behind the Casparian strip (Fig. 7*B*). This 2-step mechanism would explain why the double knockout *btsl* mutant has a mild phenotype, but a *bts* knockout is not viable. Although the *btsl* mutant takes up excess Fe in the root, BTS is able to regulate Fe redistribution in the rest of the plant. If BTSLs are functional, but BTS is not, the accumulation of toxic levels of Fe causes embryo lethality (17). The expression of *BTSL2* and *BTS* may overlap in the stele of the uptake zone where the Casparian strip is not fully suberinized (Fig. 2). It will be interesting to study the cell-specific turnover of BTSL and BTS targets, by using constitutively expressed FIT-GFP and ILR3-GFP, respectively. Moreover, the model could help explain the observed local and systemic control of Fe homeostasis (39–41).

## Materials and Methods

### Plant Material, Cultivation, and Phenotyping.

*A. thaliana* wild-type and mutant lines, growth conditions, Fe treatments, and analysis of chlorophyll and Fe concentrations are described in detail in *SI Appendix*, *SI Materials and Methods*.

### Promoter Activity and Gene Expression Analysis.

The promoter:GUS/GFP constructs and quantitative reverse transcriptase PCR are described in *SI Appendix*, *SI Materials and Methods*.

### Protein Production and Protein Interaction Studies.

Details of the constructs for protein expression in *E. coli*, protein purification, and interaction studies by co-IP and far-Western blot analysis are provided in *SI Appendix*, *SI Materials and Methods*.

All other methods, such as in-vitro ubiquitination and western-blot analysis are described in *SI Appendix*, *SI Materials and Methods*.

## Supplementary Material

Supplementary File

Supplementary File
