# Supplementary material for: Arabidopsis BRUTUS-LIKE E3 ligases negatively regulate iron uptake by targeting transcription factor FIT for recycling
Source: Proc Natl Acad Sci U S A. 2019 Aug 14;116(35):17584–91. doi: 10.1073/pnas.1907971116 (PMC6717287; doi:10.1073/pnas.1907971116)
Supplement: Supplementary File [file pnas.1907971116.sapp.pdf]

PNAS
www.pnas.org

Supplementary Information for

BRUTUS-LIKE E3 ligases negatively regulate iron uptake by targeting transcription factor FIT for recycling

Jorge Rodríguez-Celma, Robert T. Green, James M. Connorton, Inga Kruse, Yi-Tze Chen, Yan Cui, Hong Qing Ling, Kuo Chen Yeh, Janneke Balk

Corresponding author: Janneke Balk
Email: janneke.balk@jic.ac.uk

This PDF file includes:

Supplementary text (SI Materials and Methods)
Figs. S1 to S10
Tables S1 to S3
References for SI reference citations

www.pnas.org/cgi/doi/10.1073/pnas.1907971116

SI Materials and Methods

Plant Material and Growth Conditions. *Arabidopsis thaliana* ecotype Columbia (Col-0) plants were used as wild type. The T-DNA insertion lines were *btsl1-1* (SALK_015054); *btsl1-2* (SALK_032464); *btsl1-3* (SALK_117926); *btsl2-2* (SALK_048470); and *bts-1* (SALK_016526) from the Nottingham Arabidopsis Stock Centre. All primer sequences used for genotyping are listed in SI Appendix,Table S2.

Plants were grown on half Hoagland solution with 0.8% (w/v) agar. The medium was composed of (in mM): K (3.5), Mg (1), Ca (2.5), NO_3 (7.5), PO_4 (1), and SO_4 (1); and (in µM): Mn (9.14), B (46.3), Mo (0.12), Zn (2.4) and Cu (0.37). The pH was adjusted to 5.5 with 1 mM 2-(N-morpholino)ethanesulfonic acid (MES). Fe was added as FeEDTA at the indicated concentrations. To induce Fe deficiency, 100 µM of 3-(2-pyridyl)-5,6-diphenyl-1,2,4-triazine sulfonate (ferrozine) was added. For seed propagation, plants were grown on compost under long-day conditions, 16 h light at 22 ˚C, 8 h dark at 20 ˚C, with 80% humidity and a light intensity of 140 – 200 µmol m^{-2} s^{-1}.

Co-expression analysis. The co-expression networks were built using maccu software (http://maccu.sourceforge.net/) and publicly available microarray data (NASCArrays; http://affymetrix.arabidopsis.info/). Data sets from experiments labelled 'roots' were pooled for the root network, and those labelled 'leaves or shoots' for the shoot network. Networks were visualized using Cytoscape 3.0 (http://www.cytoscape.org/). *BTSL1*, *BTSL2* and *BTS* were used as baits to retrieve co-expressed genes with a Pearson correlation coefficient >0.60 for *BTSL1* and *BTSL2*, and >0.50 for *BTS* (SI Appendix, Table S3). Only those genes present in the published ferrome (1) were used for the network in SI Appendix, Fig. S2.

Promoter GUS and GFP Studies. For the *BTSL1* promoter, the whole intergenic region upstream of the ATG was used (-880 nt), and for *BTSL2* we cloned -2097 nt of the 5 kb intergenic region. The promoter fragments were cloned upstream of eGFP-GUS in pBGWFS7 (2). Glucuronidase activity was detected as described in (3), keeping the reaction time constant for equivalent samples. Seedlings were cleared in 100% ethanol, or

fixed with a solution of 3.7% (w/v) formaldehyde, 5% (v/v) acetic acid, 50% (v/v) ethanol, followed by embedding in TechnoVit (Heraeus Kulzer GmbH, Germany), per manufacturer instructions. For confocal imaging, seedlings were incubated for 30 minutes in 2.5 mg/L propidium iodide (PI) and washed with distilled water. Then, GFP and PI fluorescence was imaged using a TCS SP8X confocal microscope from Leica (Germany).

Plant Phenotyping. Chlorophyll was extracted following a modified protocol from (4). Iron content in leaves was determined by a colorimetric assay. Dried and powdered samples (4–8 mg) were mineralized in 150 µl nitric acid (65%) for 2 days and 150 µl H_2O_2 (30%) for 2 more days. The volume was adjusted to 500 µl with sterile water, and 100 µl were neutralized with 1 ml of 15% (w/v) ammonium acetate and reduced with 100 µl of 4% (w/v) ascorbic acid. Fe(II) was chelated by adding 100 µl of 1.5% w/v 3-(2-pyridyl)-5,6-bis(5-sulfo-2-furyl)-1,2,4-triazine (Ferene), and the absorbance measured at 593 nm. To localize Fe in tissues, plants were incubated with Perls' stain solution (equal volumes of 2% [v/v] HCl and 2% [w/v] K-ferrocyanide) for 30 min. Seedlings were washed three times with water, and incubated in 50% ethanol to partially clear the tissue. Fe chelate reductase activity was measured following (5).

Quantitative reverse-transcription PCR. Total RNA was extracted using the Plant RNeasy kit (Qiagen), followed by DNase treatment (Turbo DNase kit, Agilent). RNA (4 µg) was reverse transcribed to cDNA using Superscript III (Thermo Fisher). RT-qPCR reactions were made using SensiFAST master-mix (Bioline), in 20 µl volumes, each with 20 ng of cDNA. Reactions were measured in a Bio-Rad CFX-96 real-time PCR system and cycled as per the Bioline protocol. The house-keeping genes *SAND* (*AT2G28390*) and *TIP41-like* (*AT4G34270*) were used as reference genes, as they are unaffected by Fe levels in Arabidopsis (6).

Western Blot Analysis. Roots from ten plants were ground in liquid nitrogen and proteins were precipitated with acetone containing 10% (w/v) trichloroacetic acid. After centrifugation and washing the pellet with cold acetone, proteins were solubilized with 8 M urea, 0.5% (w/v) SDS, 50 mM DTT and 1 mM PMSF. Proteins were separated on 12.5%

SDS-PAGE gels, blotted into nitrocellulose membranes and detected using anti-ferritin, anti-FIT or anti-IRT1 (7) and chemiluminescence substrate. Antibodies against ferritin were produced using purified pea ferritin, and antibodies against FIT were raised against purified MBP:FIT protein, both in rabbits.

Immunoaffinity Purification and Protein Identifcation by Mass Spectrometry. The coding sequence of *FIT* was cloned into the pEarleyGate 203 vector, adding a N-terminal Myc tag and placing expression under the control of the CaMV 35S promoter, for *Agrobacterium*-mediated plant transformation. Plants carrying an integrated copy of Myc-FIT (Myc-FIT plants) and non-transformed plants as negative control, were grown in ½ Murashige and Skoog medium for 14 days. Root proteins were extracted in buffer containing 50 mM Tris-HCl pH 8.1, 250 mM NaCl, 0.5% (v/v) Triton X-100, 50 μM MG132, and 1x EDTA-free Protease Inhibitor Cocktail. Myc-FIT was immuno-precipitated using Sigma EZview Red Anti-c-Myc Affinity Gel following the manufacturer's protocol. Myc-FIT and candidate interacting proteins were eluted with loading buffer (250 mM Tris pH 6.8, 4% (w/v) SDS, 20% (v/v) glycerol, 2% (v/v) β-mercaptoethanol), boiled for 3 min, then separated by SDS-polyacrylamide gel electrophoresis (PAGE). The lane was divided in gel slices which were subjected to tryptic digestion. Peptide masses were analyzed using a LTQ-Orbitrap Velos and proteins were identified using MACOT engine. The unique peptide EAVPGQNPSYR from BTSL2 (AT1G18910.1) was found in two independent pull down experiments using roots from Myc-FIT plants, but not when using control plants. The MASCOT ion scores for the BTSL2 peptides were 16 and 25, respectively, which are over the threshold of statistical significance ($p < 0.05$).

Recombinant Protein Production. Full coding sequences of *FIT* (*AT2G28160*) and *bHLH39* (*AT3G56980*) were cloned in frame with a C-terminal Myc tag and inserted into the pET-15b vector using NdeI and BamHI restriction sites, adding a N-terminal His tag. The C-terminal coding regions of *BTSL1* (codons 933-1259) and *BTSL2* (codons 787-1254) were cloned in frame with a C-terminal Strep-tag, into the expression vector pMAL-c5X (New England Biolabs) using NotI-SalI and NcoI-SalI restriction sites, respectively. This generated also an N-terminal fusion with maltose binding protein (MBP). A construct

for MBP:FIT was produced in the same way. Plasmids were transformed into *E. coli* Rosetta strain (New England Biolabs). Cells were grown in LB and protein expression was induced with IPTG (Isopropyl β-D-1-thiogalactopyranoside) for 16 h at 16°C. The recombinant FIT and BTSLc proteins were purified using Ni-NTA (Qiagen) and Strep-tactin Sepharose (IBA), respectively, following manufacturer instructions. All primers used are listed in SI Appendix, Table S2.

Co-Immunoprecipitation Assays. Purified recombinant FIT protein was mixed with either buffer or purified BTSL1c or BTSL2c proteins and incubated for 2 h at 4°C with Strep-tactin XT® (IBA). Beads were recovered by centrifugation at 1000 x *g* at 4°C and washed 3 times with coIP-buffer (50 mM Tris HCl pH 8.0, 150 mM NaCl, 0.05% (v/v) IGEPAL and 0.07% (v/v) β-mercaptoethanol). Proteins were eluted with loading buffer (250 mM Tris pH 6.8, 4% (w/v) SDS, 20% (v/v) glycerol, 2% (v/v) β-mercaptoethanol) at 90°C for 10 minutes. The interaction was determined by western blot using anti-Myc (ab18185, Abcam) and anti-MBP (ab23903, Abcam) antibodies, respectively.

Far-Western Blot Analysis. Soluble protein extracts of bacteria expressing Myc-tagged FIT and bHLH39 proteins were separated on a 10% SDS PAGE gel and blotted to nitrocellulose membrane. Membranes were then blocked and incubated with purified MBP, MBP:FIT, MBP:BTSL1c or MBP:BTSL2c at 0.5 µM in TBS-tween buffer with 5% (w/v) dry milk as blocking agent. After washing, proteins bound to the membrane were detected with anti-Myc (ab18185, Abcam) and anti-MBP (ab23903, Abcam) antibodies, respectively.

In-vitro Ubiquitination. Assays were carried out using a ubiquitinylation kit (ENZO Life Sciences) following manufacturer's instructions. In brief, purified MBP-BTSL1c or MBP-BTSL2c (E3) and FIT (substrate) were incubated with ubiquitinylation buffer, 5 mM Mg-ATP solution, 1 mM DTT, 100 nM human E1, 2.5 mM human E2 (UbcH5b), 2.5 mM biotin-labelled ubiquitin, and 1 unit of inorganic pyrophosphatase (New England Biolabs) at 37°C for 16 h. A sample lacking Mg-ATP served as negative control. Proteins were

separated on a 7.5% (w/v) SDS-PAGE under non-reducing conditions. BTSL and FIT proteins were detected using anti-MBP and and anti-FIT antibodies, respectively.

Cell-Free Degradation Assays. Ten-day-old seedlings of type and the *btsl* double mutants were used for cell-free degradation assays as previously described (8). Briefly, an extract of whole seedlings in a proteasome compatible buffer (25 mM Tris-HCl pH 7.5, 10 mM NaCl, 10 mM $MgCl_2$, 4 mM PMSF, 5 mM DTT and 10 mM ATP) was made, and recombinant Myc-tagged FIT was added. Reactions were carried out with 160 mM MG132 or without MG132 (dimethyl sulfoxide only) at 22°C. Samples were taken at 0, 30 and 60 minutes, followed by separation on 10% (w/v) SDS PAGE gel. Immunodetection was carried out using anti-Myc antibody as described above.

Transient Protein Expression in *Nicotiana benthamiana*.

The C-terminal region of *BTSL2* (codons 787-1254) was cloned in frame with *GFP* in the expression vector pEarleyGate 103. As a negative control, the coding sequence of *GUS* was also cloned in the same way. Each construct was expressed together with Myc-FIT in pEarleyGate 203 in *Nicotiana benthamiana* using *Agrobacterium*-mediated infiltration. In brief, *Agrobacterium* cells were grown in LB, pelleted and resuspended in infiltration buffer (10 mM MES pH5.6, 10 mM $MgCl_2$, 100 μM acetosyringone), then infiltrated with a syringe in young leaves of three weeks old *Nicotiana benthamiana* plants. Five days post-infiltration, leaves were re-infiltrated with either a solution containing 50 μM MG132 or DMSO (mock treatment). Tissue was harvested after 2 h and frozen in liquid nitrogen, Proteins were extracted and separated by10% (w/v) SDS-PAGE. After transfer to a PVDF membrane, proteins were detected using anti-Myc, anti-GFP and anti-BTSL2 antibodies.

A

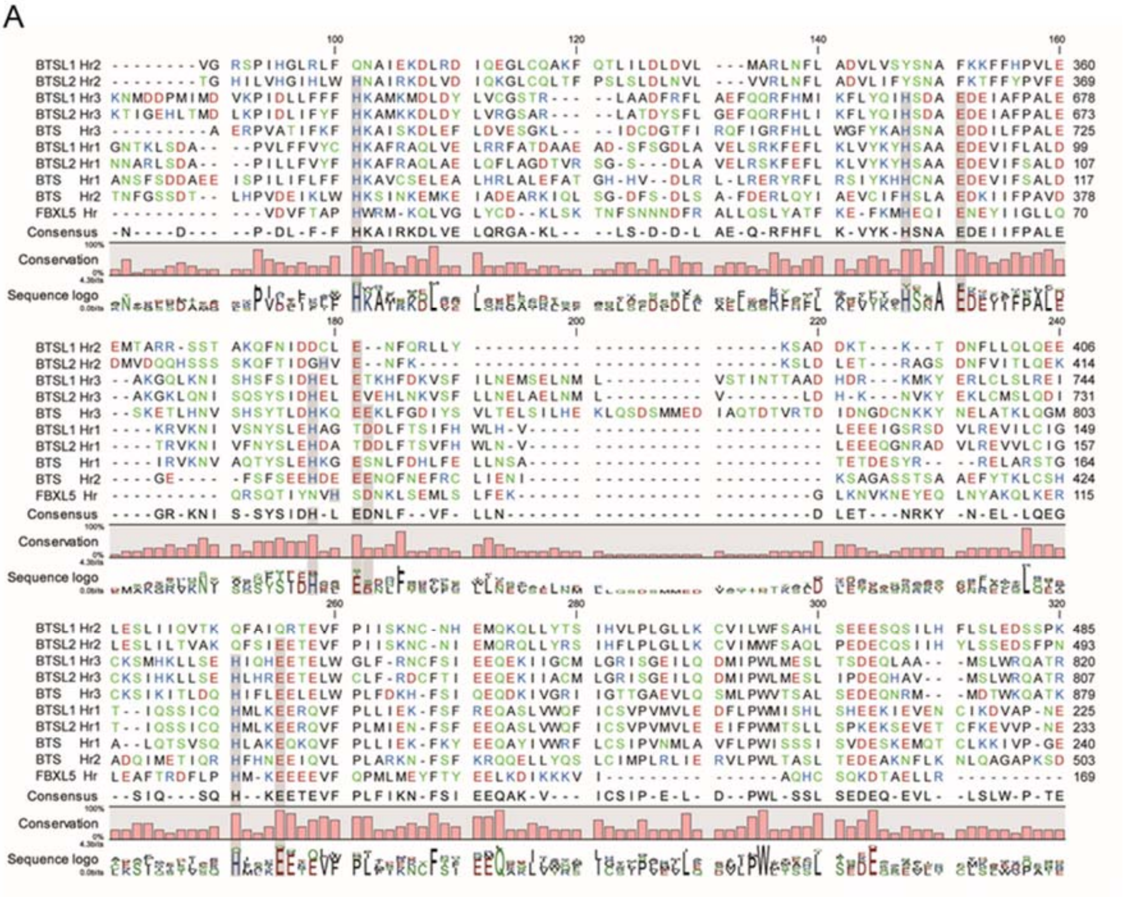

B

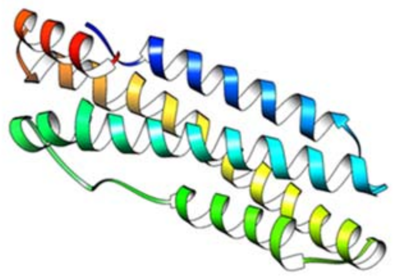

Fig. S1. Sequence analysis of hemerythrin motifs in BTSL and BTS proteins. (*A*) Amino-acid alignment of the 3 hemerythrin (Hr) motifs in BTSL, BTS proteins with the single Hr motif in FBXL5. Aminoacid sequences from Arabidopsis BTSL1 (AT1G74770, Hr1 aa 25-225, Hr2 aa 294-485, Hr3 aa 604-820), BTSL2 (AT1G18910, Hr1 aa 36-233, Hr2 aa 301-494, Hr3 aa 599-807) and BTS (ATG3G18290, Hr1 aa 42-240, Hr2 aa 304-503, Hr3 aa 659-879) were obtained from TAIR (https://www.arabidopsis.org/). FBXL5 sequence was obtained from Uniprot (Q9UKA1, Hr aa 8-169). Alignment was performed using CLC Main Workbench (Qiagen). (*B*) Predicted structure for the second Hr domain of BTSL1 (Hr2 aa 297-446) using the Phyre2 server.

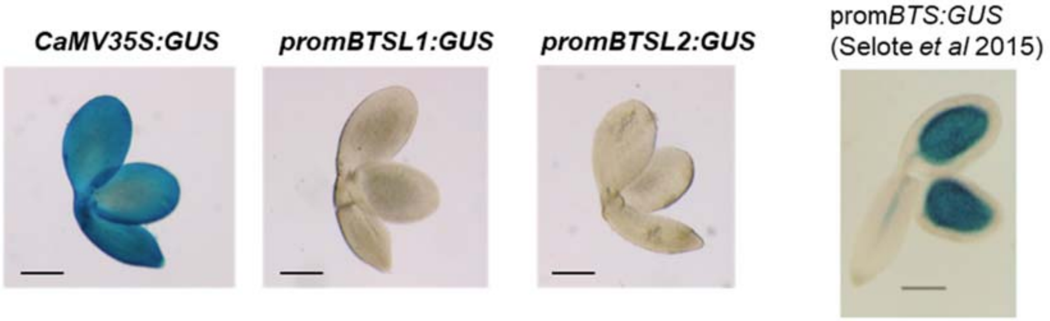

Fig. S2. Promoter activity of *BTSL1* and *BTSL2* in embryos. Promoter:*eGFP-GUS* fusions were expressed in Arabidopsis, and the embryos were dissected for staining of GUS activity. The constitutive *CaMV35S:GUS* construct served as a positive control. Data for *promBTS:GUS* are shown for comparison, taken from Selote et al., 2015. Plant Physiol 167:273–286. Scale bar 200 µm.

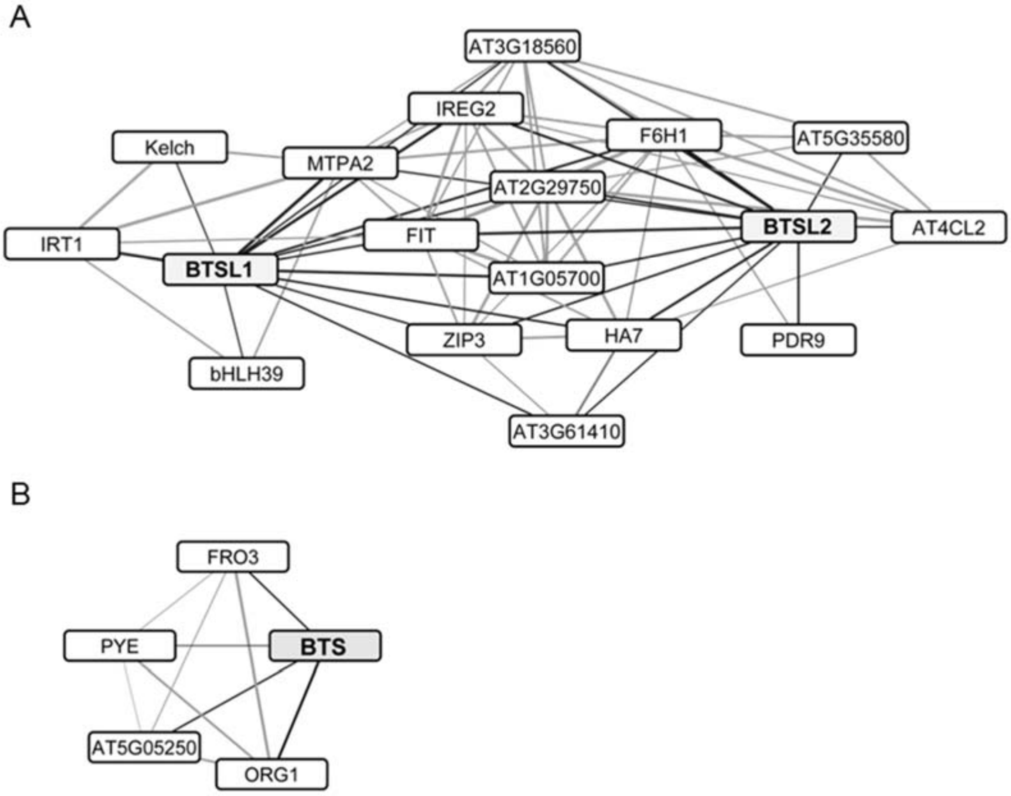

Fig. S3. *BTSL* and *BTS* are part of different gene expression networks. Co-expression analysis of Fe-responsive genes that are co-regulated with (*A*) *BTSL1* and *BTSL2* in roots, and (*B*) *BTS* in shoots. The edge thickness relate to the Pearson's correlation coefficient. See SI Appendix Table S1 for descriptions of all genes and numerical values.

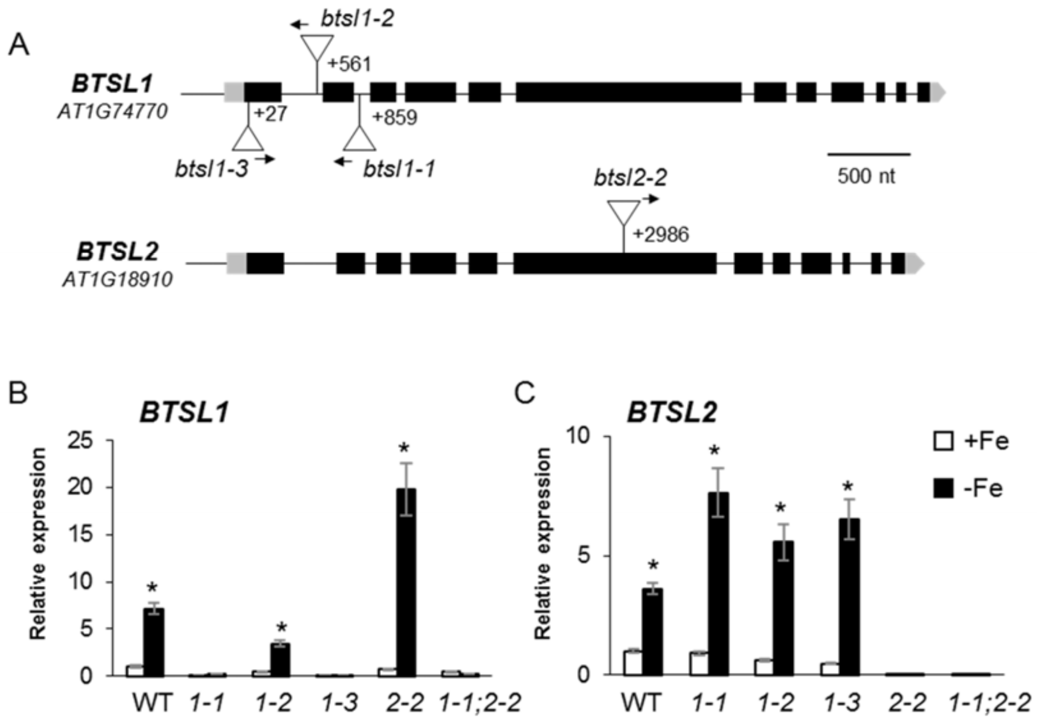

Fig. S4. *BTSL1* and *BTSL2* mutant alleles. (*A*) Schematic representation of the *BTSL1* and *BTSL2* genes and mutant alleles. Exons are in black, 5' and 3'-UTRs in grey. T-DNA insertions are indicated by triangles: *btsl1-1*, SALK_015054; *btsl1-2*, SALK_032464; *btsl1-3*, SALK_117926; *btsl2-2*, SALK_048470. Arrows indicate the left border primer used for genotyping. (*B*, *C*) Transcript levels of *BTSL1* (*B*) and *BTSL2* (*C*) in wild-type and mutant plants under standard (+Fe, 50 µM FeEDTA) and Fe-deficient conditions (-Fe, 100 µM ferrozine). RT-qPCR was carried out on biological triplicate samples.

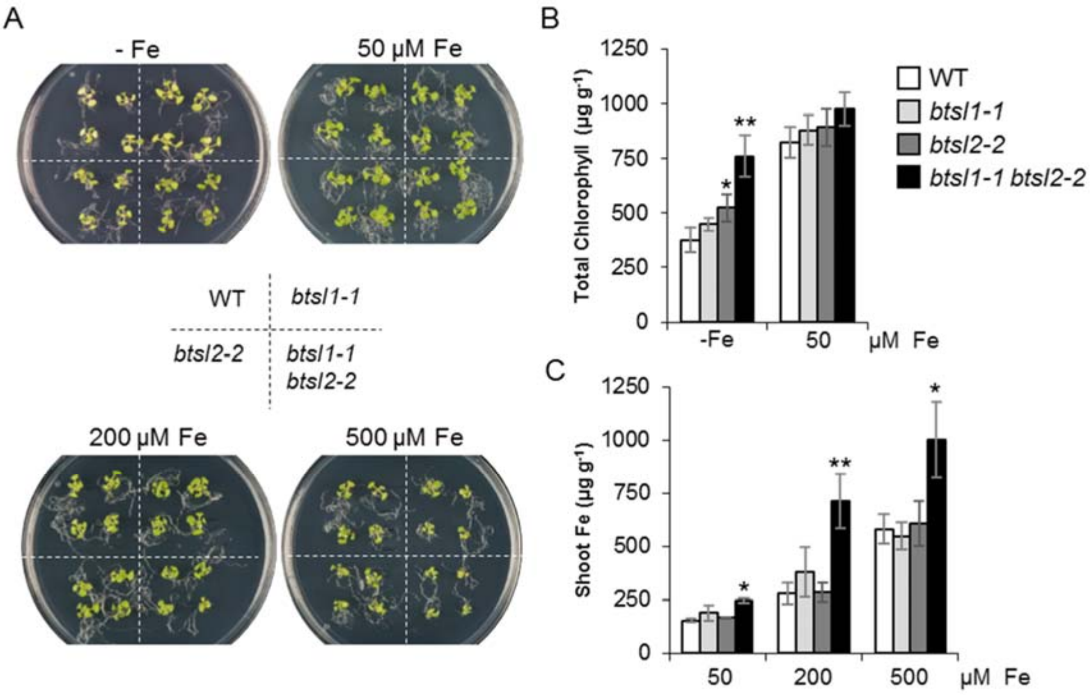

Fig. S5. Iron-associated phenotypes in mutant lines of *BTSL1* and *BTSL2*. (*A*) Growth of wild-type and *btsl* alleles on medium with varying Fe concentrations. Seeds were germinated on medium with 50 µM FeEDTA, and, after 10 days, transferred to medium depleted of Fe using 100 µM ferrozine (-Fe) or 50 µM FeEDTA; or grown in the presence of 200 or 500 µM FeEDTA for 14 days. (*B*, *C*) Chlorophyll concentration (*B*) under –Fe and control conditions, and iron concentration in shoots (*C*) from wild type (WT) and *btsl* mutant alleles, grown under control and Fe toxicity conditions as in (*A*). The bars represent the mean of n = 3 biological replicates x 10 seedlings ± SD (* $p<0.05$; ** $p<0.01$ using a two-tailed *t*-test).

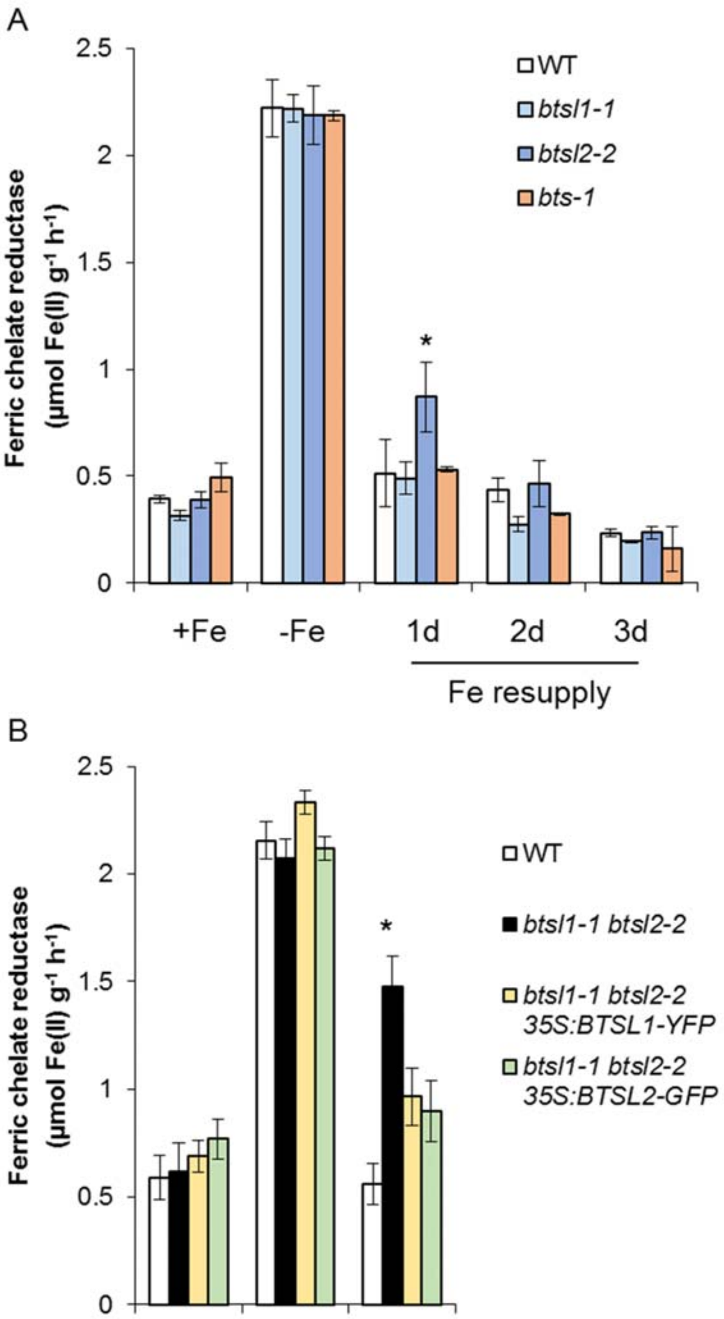

Fig. S6. Ferric chelate reductase activity in *btsl1-1*, *btsl2-2*, *bts-1* mutants and complemented lines (*A*) Ferric chelate reductase activity of *btsl1-1*, *btsl2-2* and *bts-1* single insertion mutants under the indicated Fe treatments. (*B*) Complementation of mutant alleles assessed by ferric chelate reductase activity. Wild-type, *btsl1-1 btsl2-2* double mutant and *btsl1-1 btsl2-2* lines expressing *35S:BTSL1-YFP* or *35S:BTSL2-GFP* were grown under Fe sufficiency, Fe deficiency and resupplied with Fe for 1 day. The results are the mean value of 3 biological replicates +/- SE (*, $p<0.05$ using a two-tailed Student *t*-test).

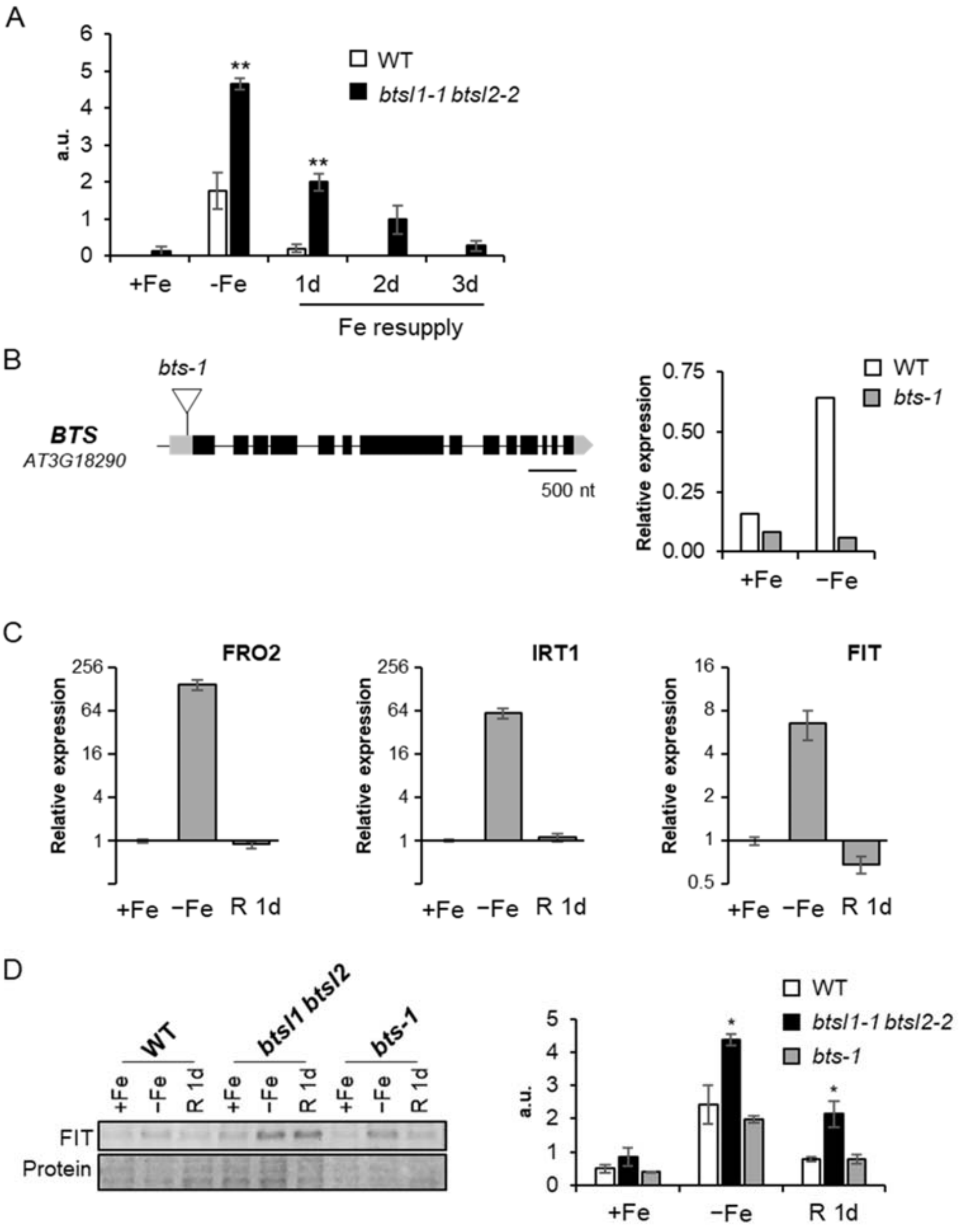

Fig S7. Mutation of *BTS* does not effect the expression of *FRO2*, *IRT1* and *FIT*, or FIT protein levels. (*A*) Quantification of FIT protein in wild type (WT) and the *btsl1-1 btsl2-2* double mutant line under Fe deficiency-resupply treatment. Values are the mean of 4 biological replicates ± SE (* $p<0.05$ using a two-tailed t-test). A representative gel is showed in Fig. 4E. (*B)* Schematic representation of the *BTS* gene and the *bts-1* mutant allele (SALK_016526). Exons are in black, 5' and 3'-UTRs in grey. The T-DNA insertion is indicated by a triangle. RT-qPCR analysis confirmed the lack of induction of *BTS* transcript upon Fe deficiency in the *bts-1* mutant, as previously shown (Selote et al., 2015). (*C*) Expression of *FRO2*, *IRT1* and *FIT* in roots from *bts-1* seedlings determined by quantitative RT-PCR. All values are relative to wild type +Fe. *(D)* Representative western-blot and bar chart of FIT protein levels in WT, *btsl1-1 btsl2-2* and *bts-1* lines in the Fe deficiency-resupply treatment. Values are the mean of 3 biological replicates ± SE (* $p<0.05$ using a two-tailed *t*-test).

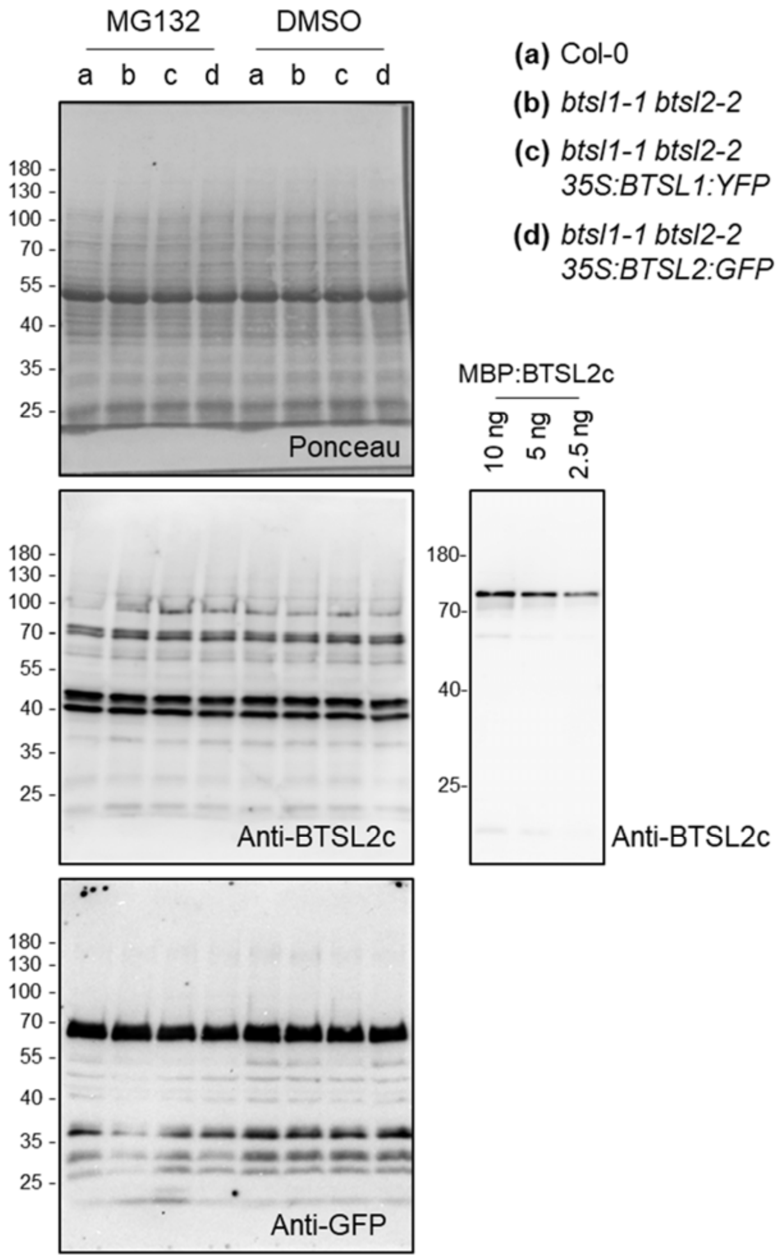

Fig S8. Protein levels of BTSL1 and BTSL2 are low. Immunoblot analysis of seedling protein extracts from wild type, *btsl* double mutant and complemented lines treated with MG132 or DMSO. Ponceau shows protein loading. Blots were developed using rabbit antibodies against the MBP:BTSL2 recombinant protein used for *in vitro* analysis. Antibodies were tested to detect ng amounts of the purified recombinant protein. Anti-GFP antibody was used to detect BTSL:GFP fusion protein. No specific signal was detected present in the wild type or complementation lines not present in the double mutant.

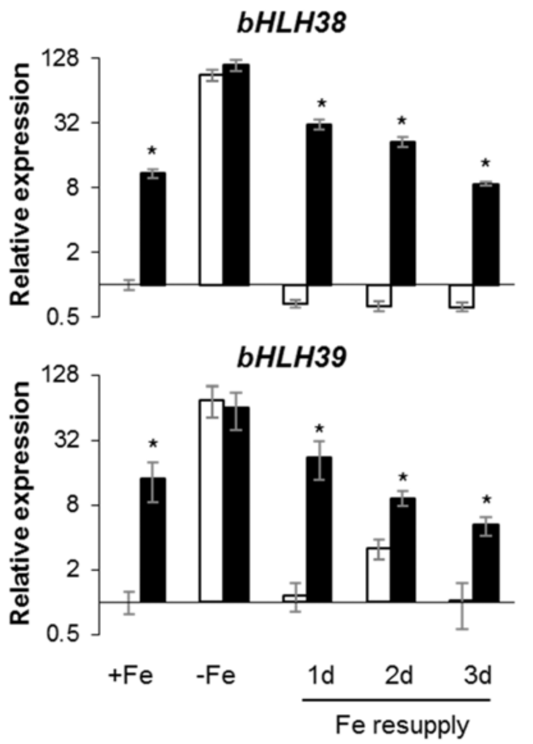

Fig. S9. Expression of *bHLH38* and *bHLH39* in roots from wild-type and *btsl1-1 btsl2-2* seedlings determined by quantitative RT-PCR. All values are relative to wild type +Fe. Values are the mean of 3 biological replicates ± SE (* $p<0.05$ using a two-tailed *t*-test).

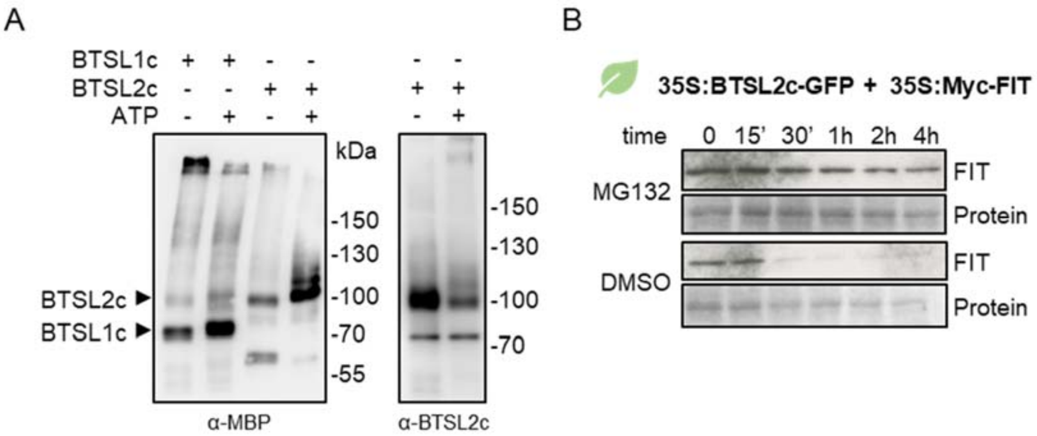

Fig. S10. E3 ubiquitin ligase activity of BTSL proteins. (*A*) In vitro self-ubiquitination activity of BTSL1c and BTSL2c in the presence of [E1 + E2] and ATP. Proteins were separated by SDS-PAGE and higher molecular weight protein products were detected by western blot analysis using anti-MBP and anti-BTSL2c antibodies. (*B*) Cell-free degradation assay in extracts from *N. benthamiana* leaves expressing Myc:FIT and BTSL2c:GFP in the presence of MG132 or DMSO. FIT protein was detected using anti-Myc antibodies and Ponceau-S staining shows equal loading.

Table S1. ATG numbers and descriptions of genes appearing in the ferrome correlation networks shown in Fig. S3.

BTSL1 and BTSL2 root ferrome network			**Correlation to**		
**ATG**	**Symbol**	**Description**	***BTSL1***	***BTSL2***	***BTS***
AT1G74770	*BTSL1*	BRUTUS-Like 1, zinc ion binding			
AT1G18910	*BTSL2*	BRUTUS-Like 2, zinc ion binding			
AT4G19690	*IRT1*	iron-regulated transporter 1	0.687		
AT3G56980	*bHLH39*	basic helix-loop-helix (bHLH) DNA-binding superfamily protein	0.607		
AT3G07720	*Kelch*	Galactose oxidase/kelch repeat superfamily protein	0.600		
AT3G53480	*PDR9*	pleiotropic drug resistance 9		0.693	
AT5G35580		Unknown		0.646	
AT3G21240	*AT4CL2*	4-coumarate:CoA ligase 2		0.628	
AT5G03570	*IREG2*	iron regulated 2	0.765	0.711	
AT1G05700		Leucine-rich repeat transmembrane protein kinase protein	0.742	0.667	
AT3G13610	*F6H1*	2-oxoglutarate (2OG) and Fe(II)-dependent oxygenase superfamily protein	0.702	0.793	
AT3G60330	*AHA7*	H(+)-ATPase 7	0.675	0.719	
AT3G58810	*MTPA2*	metal tolerance protein A2	0.664	0.643	
AT2G29750		Unknown	0.654	0.684	
AT3G61410		Unknown	0.649	0.632	
AT2G28160	*FIT*	FER-like regulator of iron uptake	0.649	0.748	
AT2G32270	*ZIP3*	zinc transporter 3 precursor	0.643	0.665	
AT3G18560		Unknown	0.626	0.659	

BTS shoot ferrome network			**Correlation to**		
**ATG**	**Symbol**	**Description**	***BTSL1***	***BTSL2***	***BTS***
AT3G18290	*BTS*	BRUTUS, zinc finger protein-related			
AT5G53450	*ORG1*	OBP3-responsive gene 1			0.734
AT1G23020	*FRO3*	ferric reduction oxidase 3			0.645
AT5G05250		Unknown			0.644
AT3G47640	*PYE*	basic helix-loop-helix (bHLH) DNA-binding superfamily protein			0.568

Table S2. List of primers.

RT-PCR

**ATG**	**Gene**	**Primer name**	**Sequence**
AT4G34270	*TIP41-LIKE*	AtTIP41F	GTGAAAACTGTTGGAGAGAAGCAA (6)
AT4G34270	*TIP41-LIKE*	AtTIP41R	TCAACTGGATACCCTTTCGCA (6)
AT2G28390	*SAND*	AtSANDF	AACTCTATGCAGCATTTGATCCACT (6)
AT2G28390	*SAND*	AtSANDR	TGATTGCATATCTTTATCGCCATC (6)
AT1G01580	*AtFRO2*	qS-FRO2F	TTACCCGATCGACCACAACAC
AT1G01580	*AtFRO2*	qA-FRO2R	CCGCACTACAAGTCGCCATTAT
AT4G19690	*AtIRT1*	qS-IRT1F	CACCATTCGGAATAGCGTTAGG
AT4G19690	*AtIRT1*	qA-IRT1R	CCAGCGGAGCATGCATTTA
AT5G01600	*AtFER1*	AtFER1F	TCGTTGAGAGTGAATTTCTGG (9)
AT5G01600	*AtFER1*	AtFER1R	ACCCCAACATTGGTCATCTG (9)
AT1G74770	*AtBTSL1*	BTSL1Fnew	GGCAATGAAGATGGATTTGG
AT1G74770	*AtBTSL1*	BTSL1Rnew	TCATATGGAACCGTTGCTGA
AT1G18910	*AtBTSL2*	BTSL2Fnew	CGGGGCAGAATCCATCTTAT
AT1G18910	*AtBTSL2*	BTSL2Rnew	GTTGCAACAAGGAGCAAGAAG
AT2G28160	*FIT*	FIT1setAF	TCGCGGGTCTTGAAGCTTCT
AT2G28160	*FIT*	FIT1setAR	GCAGGAGGATTGATACCGCG
AT3G56970	*bHLH38*	bHLH38_QA	CTGACGAAACAGATACTCCCAAGCT
AT3G56970	*bHLH38*	bHLH38_QS	TTTCACAAACTTCGGTTGGCC

Genotyping

**ATG**	**line**	**Primer name**	**Sequence**
AT1G74770	*btsl1-1*	btsl1-1_LP	TGCTTGGCATAATCCTTCTTG
AT1G74770	*btsl1-1*	btsl1-1_RP	GAACTCTCTTGCTTCCTGAAGC
AT1G74770	*btsl1-2*	btsl1-2_LP	GACTATCGTCAAGCAGCCAAG
AT1G74770	*btsl1-2*	btsl1-2_RP	GATGCTCCGGTTCTTTTCTTC
AT1G74770	*btsl1-3*	btsl1-3_LP	GGCTGACTCAGCTAATCTCCC
AT1G74770	*btsl1-3*	btsl1-3_RP	TGCAACACGAATTTAGACAAGG
AT1G18910	*btsl2-2*	bts2-2_LP	TCGGTTATTCAGGCAAAACAC
AT1G18910	*btsl2-2*	bts2-2_RP	CCCTTTGTACTCATCAGCAGC
AT3G18290	*bts-1*	bts-1_LP	CCAAATGCGTTCGTAGGTAAG
AT3G18290	*bts-1*	bts-1_RP	TCAGATTTACACAAATTTGCAGC

Promoter-GUS

**ATG**	**Promoter**	**Primer name**	**Sequence**
AT1G74770	*BTSL1*	BTSL1prom_GW F	GGGGACAAGTTTGTACAAAAAAGCAGGCT AGTATTGATTTTGTGAGCCCAATTAAC
AT1G74770	*BTSL1*	BTSL1prom_GW R	GGGGACCACTTTGTACAAGAAAGCTGGGT TCCACCGCAACAACAATAACGGTG
AT1G18910	*BTSL2*	BTSL2pro_GWF	GGGGACAAGTTTGTACAAAAAAGCAGGCT AAATTCATATAAGAATTGCTTTATAC
AT1G18910	*BTSL2*	BTSL2pro_GWR	GGGGACCACTTTGTACAAGAAAGCTGGGT CCACCATGAACAGGAGAGGAGGTG

BTSL-GFP

**ATG**	**Gene**	**Primer name**	**Sequence**
AT1G74770	*BTSL1*	BTSL1_B1-F	GGGGACAAGTTTGTACAAAAAAGCAGGCT TGATGGGAGGCGGAAA
AT1G74770	*BTSL1*	BTSL1_B1-R	GGGGACCACTTTGTACAAGAAAGCTGGGT TAAGGAGCCTTGAGTT
AT1G18910	*BTSL2*	BTSL2_B1-F	GGGGACAAGTTTGTACAAAAAAGCAGGCT TGATGGGAGTCGGAGA
AT1G18910	*BTSL2*	BTSL2_B1-R	GGGGACCACTTTGTACAAGAAAGCTGGGT TGAAAAGTCTGGTGTT

Recombinant protein production

**ATG**	**Gene**	**Primer name**	**Sequence**
AT2G28160	*FIT*	FITmyc_F	ACTCATATGGAAGGAAGAGTCAACGCTC
AT2G28160	*FIT*	FITmyc_R	CGCGGATCCCTACAGATCTTCTTCAGAAATA<br>AGTTTTTGTTCAGTAAATGACTTGATGAATT<br>AAAACC
AT3G56980	*bHLH39*	39myc_F	ACTCATATGTGTGCATTAGTACCTCCATTG
AT3G56980	*bHLH39*	39myc_R	CGCGGATCCCTACAGATCTTCTTCAGAAATA<br>AGTTTTTGTTCTATATATGAGTTTCCACATTC<br>CTCATAC
AT1G74770	*BTSL1*	MBP-1CT_F	ACTCCATGGCTCAGAAGGTTAGCCAGTTTG
AT1G74770	*BTSL1*	MBP-1CT_R	TGAGTCGACTTATTTTTCGAACTGCGGGTGG<br>CTCCAAAGGAGCCTTGAGTTGTAGGAG
AT1G18910	*BTSL2*	MBP-2CT_F	ACTCCATGGAATCTTTGATCCCTGATGAAC
AT1G18910	*BTSL2*	MBP-2CT_R	TGAGTCGACTTATTTTTCGAACTGCGGGTGG<br>CTCCAGAAAAGTCTGGTGTTGTAGGAGGC

References

1. Schmidt W, Buckhout TJ (2011) A hitchhiker's guide to the *Arabidopsis* ferrome. *Plant Physiol Biochem* 49:462–470.
2. Karimi M, Inze D, Depicker A (2002) GATEWAY vectors for *Agrobacterium*-mediated plant transformation. *Trends Plant Sci* 7:193–195.
3. Jefferson RA, Kavanagh TA, Bevan MW (1987) GUS fusions: beta-glucuronidase as a sensitive and versatile gene fusion marker in higher plants. *EMBO J* 6(13):3901–7.
4. Abadía J, Abadía A (1993) Iron and plant pigments. *Iron Chelation in Plants and Soil Microorganisms*, eds Barton LL, Hemming BC (Acedmic Press, San Diego), pp 327–343.
5. Yi Y, Guerinot ML (1996) Genetic evidence that induction of root Fe(III) chelate reductase activity is necessary for iron uptake under iron deficiency. *Plant J* 10:835–844.
6. Han B, Yang Z, Samma MK, Wang R, Shen W (2013) Systematic validation of candidate reference genes for qRT-PCR normalization under iron deficiency in Arabidopsis. *BioMetals* 26:403–413.
7. Fan H, et al. (2014) SKB1/PRMT5-mediated histone H4R3 dimethylation of Ib subgroup bHLH genes negatively regulates iron homeostasis in *Arabidopsis thaliana*. *Plant J* 77:209–221.
8. Selote D, Samira R, Matthiadis A, Gillikin JW, Long TA (2015) Iron-binding E3 ligase mediates iron response in plants by targeting basic Helix-Loop-Helix transcription factors. *Plant Physiol* 167:273–286.
9. Bournier M, et al. (2013) *Arabidopsis* Ferritin 1 (*AtFer1*) gene regulation by the phosphate starvation response 1 (AtPHR1) transcription factor reveals a direct molecular link between iron and phosphate homeostasis. *J Biol Chem* 288(31):22670–22680.
